# Design, Synthesis and Structure-Activity Relationship of Novel Aphicidal Mezzettiaside-Type Oligorhamnosides and Their Analogues

**DOI:** 10.3390/molecules23010041

**Published:** 2017-12-26

**Authors:** Hui Zhao, Zhuwen Wei, Zhiyan Jiang, Sumei Li, Yixian Liao, Yiming Guo, Yongmei Tang, Weihao Chen, Guohua Zhong, Gaopeng Song

**Affiliations:** 1 College of Materials and Energy, South China Agricultural University, Guangzhou 510642, China; totom2008@163.com (H.Z.); yiyao20140401@126.com (Z.W.); cjjwcf@163.com (Y.L.); m18819262194_1@163.com (Y.G.); yanxi_2021@163.com (Y.T.); 13555660468@163.com (W.C.); 2 Key Laboratory of Crop Integrated Pest Management in South China, Ministry of Agriculture, Guangzhou 510642, China; zyjiang@stu.scau.edu.cn; 3 Lab of Insect Toxicology, South China Agricultural University, Guangzhou 510642, China; 4 Department of Human anatomy, School of Medicine, Jinan University, Guangzhou 510632, China; lisumei1234@163.com

**Keywords:** mezzettiaside, rhamnolipid, synthesis, aphicidal activity, structure–activity relationships

## Abstract

Oligosaccharides have been used for an environmentally friendly insect control in the agricultural industry. In order to discover novel eco-friendly pesticides, a series of partially acetylated oligorhamnoses mezzettiasides, **2**–**8**, and their analogues, **9**–**14**, with biosurfactant characteristics were designed and synthesized, some of which exhibited comparable to or even stronger aphicidal activity than pymetrozine. Preliminary SAR studies demonstrated that the aphicidal activity of mezzettiasides analogs is highly dependent on their structures, including both the sugar length and the substitutes on the sugar. Among them, trirhamnolipid **9** displayed the strongest aphicidal activity, with an LC_50_ of 0.019 mmol/L, indicating that the biosurfactant **9** may have potential for use as an environmentally friendly agricultural pesticide.

## 1. Introduction

Currently, the emergence of insect resistance and other negative side effects due to indiscriminate use of insecticides have limited the utility of many established classes of chemicals [1,2]. Hence, there is an urgent task to discover novel pest control agents with a new mode of action as alternatives to conventional insecticides. Oligosaccharides, a class of important bioactive molecules, play an important role in biological systems including cell adhesion, cell–cell interaction, and immunogenic recognition [3,4]. Most of them exhibited various pharmacological effects, such as fungicidal, antibacterial, and insecticidal activities [5,6,7]. Glycolipids with one or more acyl groups in the oligosaccharides molecule have been used for insect control in the agricultural industry as they are generally more biodegradable and more environmentally friendly than synthetic chemicals [8,9,10].

Rhamnose-containing oligosaccharides and their derivatives are widely distributed in natural products, which have received considerable attention in the agricultural industry due to the increased understanding of their wide spectrum of pharmacological effects, including potential antimicrobial and antifungal activities against plant pathogens [11,12,13]. Notably, it was found that rhamnolipids, as a partially acylated simple rhamnoside derivatives, had significant insecticidal activity [14]. For example, dirhamnolipid (**1**), containing α-1, 2-linked di-L-rhamnopyranosyl moiety and 3-hydroxydecanoic acid (3OH-C_10_ fatty acid) as well as 3-hydroxydodecanoic acid (3OH-C_12_ fatty acid), showed good insecticidal activity against aphids (*Myzus persicae*), which was isolated from diesel oil-degrading *Pseudomonas* sp. EP-3 [14].

**Figure 1:**
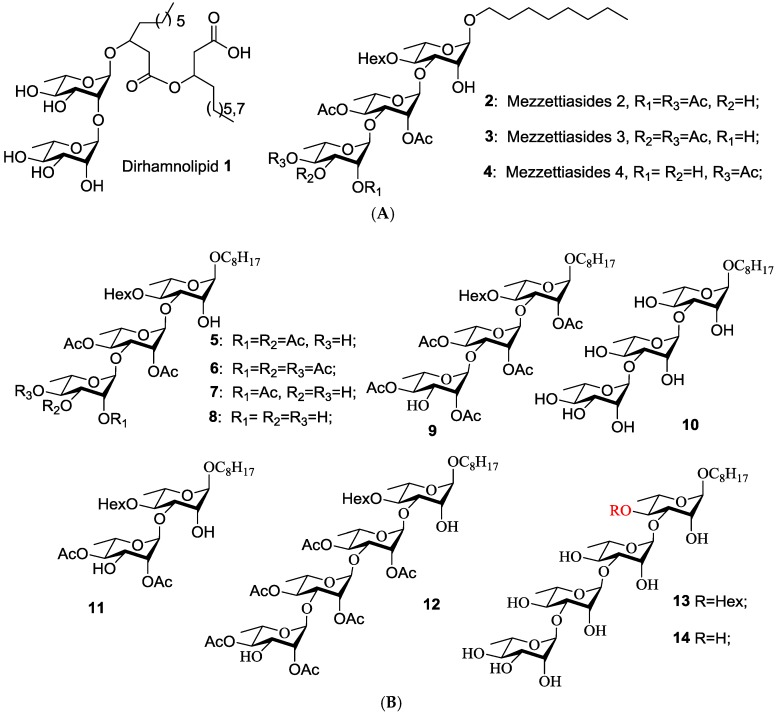
(**A**) Structures of dirhamnolipid **1** and Mezzettiasides **2**–**4**; (**B**) Structures of designed title compounds **5**–**14**.

The mezzettiasides **2**–**4** are a family of partially acetylated oligorhamnose natural products that consist of α-1,3-linked L-rhamno-tri-saccharides with different patterns of acetylation (Figure 1A), which exhibited good antibacterial and cytotoxic activity in vitro [15,16]. Structurally, mezzettiasides **2**–**4** possess biosurfactant characteristics like dirhamnolipid (**1**) and other glycolipids, such as sucrose esters, because of their molecular structures that comprise a hydrophilic portion (rhamnose moiety) and a hydrophobic portion (ester moiety). It is worth noting that some partially acylated sucrose esters display much stronger insecticidal activity when compared to sucrose itself [9,10], suggesting acylation at critical positions of oligosaccharides is helpful to enhance efficient insecticidal activity. Access to the mezzettiaside family of natural products allows us to expand the application in agriculture, as no biological data in agriculture were reported for the trisaccharide mezzettiasides **2**–**4**.

Over the years, we have been interested in the synthesis and study of partially acylated rhamnoside natural products [17]. Since compound **1** and sucrose esters were reported to possess strong insecticidal activity, the structurally novel mezzettiasides **2**–**4** with potential biosurfactant characteristics inspired us to investigate the synthesis and biological evaluation of insecticidal activity of mezzettiasides **2**–**4**. In addition, the observed variety of insecticidal activity indicated that both the length and number of acyl groups on different alcoholic hydroxyl groups seem to have important influence on insecticidal activity and the acyl groups, maybe playing a key role on in the underlying mechanism of action [18,19]. Thus, to elucidate the contribution of the acetyl groups on insecticidal activity and also to further understand the correlation between the sugar chain length and insecticidal activity, a series of rhamnolipids analogs **5**–**14** (Figure 1B) were designed and synthesized to investigate structure–activity relationships (SARs). The results obtained provide new clues for the understanding of their insecticidal profile for these types of oligosaccharides.

## 2. Results and Discussion

### 2.1. Synthesis and Characterization

Over the years, we have been interested in the synthesis and biological evaluation of partially acylated rhamnose-containing oligosaccharides via a traditional carbohydrate approach [17,20]. Several acetyl groups in mezzettiasides (di-, tri-, and tetrasaccharides) require attention in the convergent synthesis due to their O to O migration in some acidic or basic conditions. Based on our previous work [17,20], we chose isopropylidene and levulinoyl groups as hydroxy protecting groups, which could be selectively removed without affecting acetyl and hexanoyl groups under mild conditions. Furthermore, the glycosylation could provide the pure 1,2-trans-glycoside when C-2-OH in the glycosyl donor was protected with participating group such as Ac or Lev groups [4,17]. Notably, exclusive α-stereoselectivity in the glycosylation of rhamnopyranosyl donors that were either glycosylated at C-2 or blocked at this position with a non-participating group, such as the isopropylidene group, have been reported on several occasions in the literature [4,17].

**Scheme 1:**
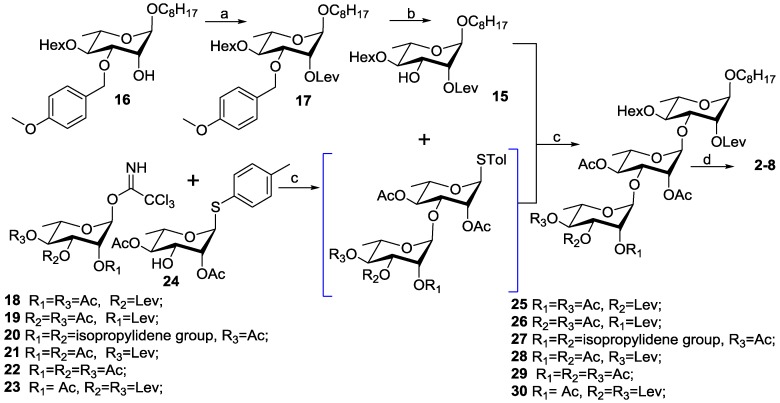
Synthesis of the title compounds **2–8**. *Reagents and conditions:* (**a**) LevOH, EDC•HCl, DMAP, CH_2_Cl_2_, 92%; (**b**) DDQ, CH_2_Cl_2_-H_2_O, 94%; (**c**) (i) TMSOTf, 4 Å MS, −78 °C, CH_2_Cl_2_; (ii) NIS/AgOTf, 0 °C, 86% for **25**, 88% for **26**, 83% for **27**, 88% for **28**, 89% for **29**, 84% for **30**; (**d**) AcOH•NH_2_NH_2_, MeOH–CH_2_Cl_2_, r.t. 5 h for **2** (88%), **3** (85%), and **5** (87%), **6** (88%), **7** (78%); AcOH•NH_2_NH_2_, MeOH–CH_2_Cl_2_, 40 °C, 18 h for **8**, 71%; (i) AcOH•NH_2_NH_2_, MeOH–CH_2_Cl_2_, r.t. 5 h, (ii) 80% AcOH for **4**, 78% for two steps.

As shown in Scheme 1, the seven mezzettiasides **2**–**8** could be efficiently prepared from coupling of the key monosaccharide building block **15** with appropriate glycosyl donors First of all, L-rhamnopyranosyl residue **16** [20] was prepared following our previous procedure, starting from readily available L-rhamnose. Treating **16** with levulinic acid (LevOH) in the presence of 1-(3-dimethylaminopropyl)-3-ethylcarbodiimide hydrochloride (EDC•HCl) and DMAP afforded **17**, and then following deprotection of the 3′-*O*-PMB group with 2,3-dichloro-5,6-dicyano-1,4-benzoquinone (DDQ) furnished the important intermediate **15**. Then, different trichloroacetimidates **18**–**23** [20,21] and the key monosaccharide thioglycoside **24** [21] were obtained following a similar strategy as that developed by us. Of special note, trisaccharides **25**–**30** were constructed with exclusive α-glycosidic linkage in 83–89% good yield via a ‘one-pot sequential glycosylation’ strategy, utilizing different trichloroacetimidates **18**–**23** and the key monosaccharide thioglycoside **24** as sequential glycosyl donors for two glycosidic linkages [22]. The α-configuration of the newly formed glycosidic bond in compound **27** was confirmed by HMBC spectrum (^1^*J*_C-1_’’’, _H-1_’’’ = 171.8 Hz). Deprotection of the Lev group in **25**–**30** with hydrazine acetate at r.t. for 5 h or at 40 °C for 18 h afforded **2**–**3**, and **5**–**8**, followed by removal of the isopropylidene group, led to **4**.

**Scheme 2:**
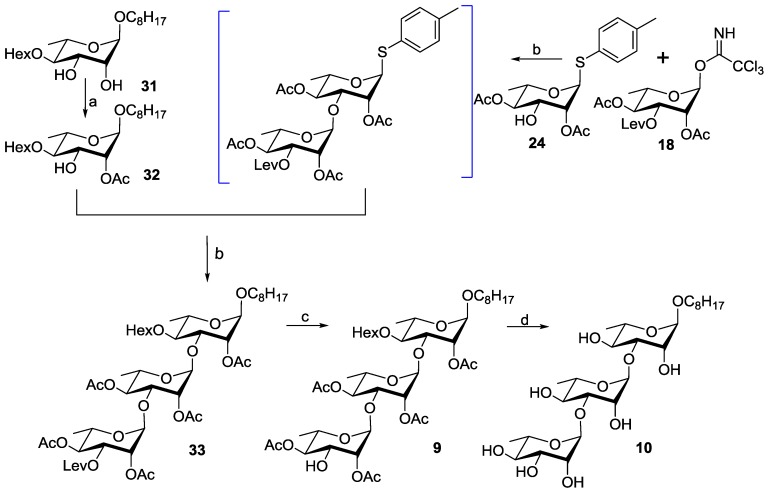
Synthesis of the title compounds **9–10**. *Reagents and conditions:* (**a**) (i) MeC(OEt)_3_, CSA, DMF, (ii) 1 M HCl, 86% for two steps; (**b**) (i) TMSOTf, 4 Å MS, −78 °C, CH_2_Cl_2_; (ii) NIS/AgOTf, 0 °C, 82% for two steps; (**c**) AcOH•NH_2_NH_2_, MeOH–CH_2_Cl_2_, r.t. 3 h, 90%; (**d**) MeONa, CH_3_OH–CH_2_Cl_2_, 86%.

The next step was to elaborate title compounds **9** and **10**. As shown in Scheme 2, treatment of the known compound **31** [20] with triethylorthoacetate and a catalytic amount of (1S)-(+)-camphor-10-sulfonic acid (CSA) formed the corresponding orthoesters, and then the reaction mixture was diluted with dichloromethane and shaken with 1 mol·L^−1^ HCl solution to furnish **32**. Trisaccharide **33** was obtained according to a similar procedure as that of **25**, followed by removal of the Lev group yielding **9**. Deprotection of the Hex and Ac groups in **9** with NaOMe in MeOH gave the target compound **10**.

**Scheme 3:**
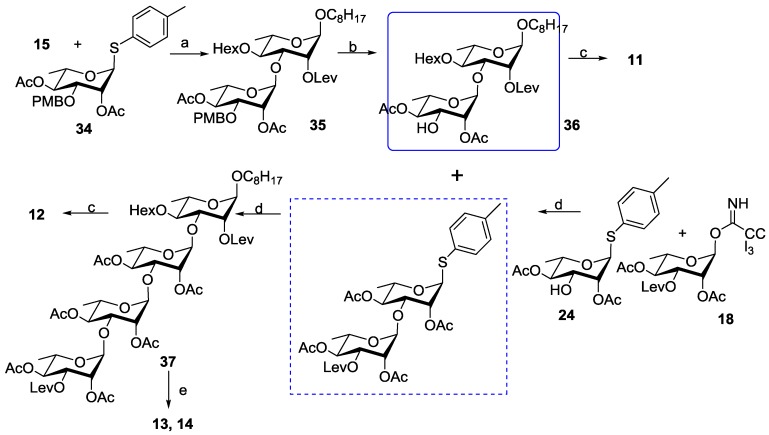
Synthesis of the title compounds **11–14**. *Reagents and conditions*: (**a**) NIS/AgOTf, 4 Å MS, CH_2_Cl_2_, 92%; (**b**) DDQ, CH_2_Cl_2_–H_2_O, 94%; (**c**) AcOH•NH_2_NH_2_, MeOH–CH_2_Cl_2_, r.t. 3 h, 89%; (**d**) (i) TMSOTf, 4 Å MS, −78 °C, CH_2_Cl_2_; (ii) NIS/AgOTf, 0 °C, 80%; (**e**) CH_3_ONa, CH_3_OH, 0 °C, 2 h for **13**, 82%; CH_3_ONa, CH_3_OH, 50 °C, 24 h for **14**, 91%.

We then turned our attention to the synthesis of the final required protected **11** and **12** from the remaining pivotal intermediate **15** (Scheme 3). The key disaccharide **35** was obtained by condensation of acceptor **15** with glycosyl donor **34** [20], followed by cleavage of 3′-*O*-PMB group with DDQ led to compound **36**. As before, a selective deprotection of the Lev group using hydrazine acetate generated **11**. With glycosyl acceptor **36** and compound **18** as well as **24** in hand as sequential glycosyl donors for two glycosidic linkages, glycosylations were performed to provide the tetrasaccharide **37**, followed by deprotection of the Lev group with hydrazine acetate yielded **12**. Selective deprotection of all the Ac groups in **12** was successfully achieved using MeONa in MeOH at 0 °C for 2 h to get **13**, of which Hex group was removed at 50 °C for 24 h to produce the title compound **14**.

### 2.2. Aphicidal Activity and Structure–Activity Relationships *Against A. glycines*

The aphicidal activities of the synthesized oligosaccharides **2**–**14** against *A. glycines* were used to guide structure–activity optimization. The widely used commercial insecticide pymetrozine was used as a reference standard. All of the compounds were initially tested at a concentration of 100 mmol·L^−1^ and consequently the compounds with high insecticidal potency were investigated further at low concentration.

As shown in Table 1, the preliminary bioassay results indicated that all the oligo-rhamnoside analogues led to different aphid mortality at 100 mmol·L^−1^ after 48 h, which shows clearly that the aphicidal activity of the oligosaccharides **2**–**14** is highly sensitive to the change of their structures. On the basis of the primary experimental results, oligo-rhamnosides displaying a mortality rate higher than 50% (at 100 mmol·L^−1^, 48 h) were chosen to determine the LC_50_ values. Among these analogues, compounds **2**, **7**, and **9** exhibited comparable to or even stronger aphicidal activity than pymetrozine. Notably, compound **9** showed the strongest aphicidal activity with an LC_50_ of 0.019 mmol/L, indicating that it could be selected as the most promising candidate for further structure modification.

Based on the aphicidal data, the preliminary structure–activity relationship (SAR) for these oligo-rhamnoside products **2**–**14** was elucidated. Comparing the activity of the disaccharide **11** with the more complex trisaccharide **2**, we saw only a small decrease in activity for **11**. However, potency did not improve with further increased chain length when moving from the trisaccharides **2**–**10** to the tetrasaccharide mezzettiasides **12**–**14**. These results revealed the disaccharide residue 1-*O*-*n*-Octyl-*α*-l-rhamnopyranosyl-(1→3)-*α*-l-rhamnopyranoside played a critical role in bioactivity. The decreased aphicidal activity of compound **10,** compared with another trisaccharide analogues **2**–**9**, indicated subtle modifications of rhamnosyl moiety with acetyl group was beneficial to enhance the insecticidal activity. A similar trend was found for the tetrasaccharides since tetrasaccharide **12** was the more active than other two tetrasaccharide products **13**–**14**. Structurally, oligo-rhamnosides **2**–**14** possess biosurfactant characteristics like **1**, including both a lipophilic and hydrophilic moiety. Thus, we hypothesized that acetylation of the different hydroxyl groups in the sugar residues could significantly affect the biosurfactant property of the above oligo-rhamnosides, which determines the interaction with some chemicals of the aphid membranes or a different mode of action. However, while there is not enough information to fully understand the effects of the acetate groups on the aphicidal activity, the location of the individual acetate groups has a significant impact on the bioactivity. For instance, introduction of either the Ac group or the l-rhamnose chain at the *C*-3-OH in the terminal rhamnose sugar of **2** led to a significant decrease in the aphicidal activity, revealing that the *C*-3-OH group in the terminal rhamnose sugar might play an essential role in the aphicidal activity, whereas either esterification or etherification of *C*-3-OH group should be avoided. In contrast, the testing of compounds **2** and **9** clearly demonstrates the importance of the modification of *C*-2-OH group in the first rhamnose sugar with Ac group. It was found that the removal of the acetate of the terminal sugar in mezzettiaside-2 (**2**) had a large negative effect on its potency. Furthermore, the simple migration of a *C*-4-Ac group on the terminal sugar in **4** to the C-2 position in **7** dramatically enhanced its aphicidal activity. Therefore, it can be concluded that in all three types of mezzettiasides (di-, tri-, and tetrasaccharides) a strong dependency upon the degree and location of acylation on the aphicidal activity can be seen. Microscopy analyses of aphids treated with dirhamnolipid (**1**) revealed that compound **1** caused insect death by affecting cuticle membranes [14]. We hypothesized that oligo-rhamnosides **2**–**14** with biosurfactant characteristics like **1** maybe possess a similar mode of action against aphicidal activity as **1**, which are currently being tested and these will be reported in due course.

## 3. Materials and Methods

### 3.1. General Methods

Solvents were purified in a conventional manner. Thin layer chromatography (TLC) was performed on precoated E. Merck silica gel 60 F254 plates. Flash column chromatography was performed on silica gel (200–300 mesh, Qingdao, China). ^1^H-NMR and ^13^C-NMR spectra were taken on a JEOL JNM-ECP 600 spectrometer with tetramethylsilane as an internal standard, and chemical shifts are recorded in ppm values. Mass spectra were recorded on a Q-TOF Global mass spectrometer.

### 3.2. Chemical Synthesis

#### 3.2.1. 1-Octyloxy-4-*O*-Hexanoyl-3-*O*-(P-Methoxybenzyl)-2-*O*-Levulinoyl-Α-l-Rhamno-Pyranoside (**17**)

To a solution of the known compound **16** (7.2 g, 14.8 mmol) in dry dichloromethane (120 mL), levulinic acid (12.2 g, 18.6 mmol), 1-(3-Dimethylaminopropyl)-3-ethylcarbodiimide hydrochloride (4.2 g, 21.6 mmol), and 4-dimethylaminopyridine (0.36 g, 2.9 mmol) were added under argon. The mixture was stirred for 48 h, diluted with CH_2_Cl_2_ (200 mL), washed 1 mol·L^−1^ HCl (2 × 100 mL), satd aq NaHCO_3_ (2 × 100 mL), and brine (2 × 100 mL), dried over Na_2_SO_4_, and concentrated under reduced pressure. The residue was purified by silica gel column chromatography (8:1, petroleum ether—EtOAc) to yield **17** (4.0 g, 92%). ^1^H-NMR (CDCl_3_): *δ* 7.18 (d, 2H, *J* = 8.6 Hz, Ar-H), 6.84 (d, 2H, *J* = 8.6 Hz, Ar-H), 4.83 (dd, 1H, *J* = 3.4, 1.9 Hz, H-2’), 4.99 (t, 1H, *J* = 9.8 Hz, H-4′), 4.72 (d, 1H, *J* = 1.6 Hz, H-1′), 4.54 (d, 1H, *J* = 11.6 Hz, Ar-CH_2_-1), 4.32 (d, 1H, *J* = 11.6 Hz, Ar-CH_2_-2), 3.80 (dd, 1H, *J* = 9.6, 3.2 Hz, H-3′), 3.74–3.76 (m, 1H, H-5′), 3.60–3.65 (m, 1H, H-1-1), 3.36–3.41 (m, 1H, H-1-2), 2.63–2.75 (m, 4H, 2 × COCH_2_), 2.24–2.27 (m, 2H, COCH_2_), 2.17 (s, 3H, COCH_3_), 1.56–1.62 (m, 4H, 2 × CH_2_), 1.30–1.33 (m, 14H, 7 × CH_2_), 1.18 (d, 3H, *J* = 6.2 Hz, H-6′), 0.90 (t, 3H, *J* = 7.1 Hz, CH_3_), 0.89 (t, 3H, *J* = 7.1 Hz, CH_3_); ^13^C-NMR (CDCl_3_): *δ* 206.4, 172.7, 171.9, 159.2, 130.1, 129.3 (two), 113.5 (two), 97.6 (Rha-C-1), 74.4. 70.8, 68.0, 66.4, 55.2, 38.1, 34.4, 31.8, 31.3, 29.8, 29.4, 29.3, 29.2, 28.3; 26.0, 24.5, 22.7, 22.3, 17.5, 14.1, 13.8; HRESIMS calcd for C_33_H_52_O_9_Na [M + Na]^+^ 615.3509; found, 615.3520.

#### 3.2.2. 1-Octyloxy-4-*O*-hexanoyl-2-*O*-levulinoyl-α-l-rhamnopyranoside (**15**)

To a stirred mixture of **17** (3.6 g, 6.0 mmol) in CH_2_Cl_2_ (54 mL) and H_2_O (6 mL), 2,3-dichloro-5,6-dicyano-1,4-benzoquinone (DDQ, 2.1 g, 9.0 mmol) was added at room temperature. The reaction was stirred for 12 h until the reaction was complete as judged by TLC. The reaction mixture was poured into saturated aqueous NaHCO_3_ (50 mL) and extracted with CH_2_Cl_2_ (2 × 150 mL). The combined organic phase was washed with satd aq NaHCO_3_ (2 × 150 mL), and brine (2 × 150 mL), dried over Na_2_SO_4_, and concentrated in vacuo. The residue was purified by silica gel column chromatography (5:1, petroleum ether–EtOAc) to provide **15** (2.7 g, 94%). ^1^H-NMR (CDCl_3_): *δ* 5.13 (dd, 1H, *J* = 3.4, 1.8 Hz, H-2′), 4.85 (t-like, 1H, *J* = 9.7 Hz, H-4′), 4.72 (d, 1H, *J* = 1.4 Hz, H-1′), 4.00 (d, 1H, *J* = 9.2 Hz, H-3′), 3.78–3.83 (m, 1H, H-5′), 3.66 (dt, 1H, *J* = 9.5, 6.6 Hz, H-1-1), 3.42 (dt, 1H, *J* = 9.6, 6.5 Hz, H-1-2), 2.81 (t, 2H, *J* = 6.4 Hz, COC*H*_2_CH_2_CO), 2.67 (t, 2H, *J* = 6.4 Hz, COCH_2_CH_2_CO), 2.37 (td, 2H, *J* = 7.2, 3.7 Hz, COCH_2_), 2.21 (s, 3H, CH_3_CO), 1.63–1.68 (m, 2H, CH_2_), 1.55–1.61 (m, 2H, H-2), 1.29–1.34 (m, 14H, 7 × CH_2_), 1.20 (d, 3H, *J* = 6.4 Hz, H-6′), 0.90 (t, 3H, *J* = 7.0 Hz, CH_3_), 0.88 (t, 3H, *J* = 7.1 Hz, CH_3_); ^13^C-NMR (CDCl_3_): *δ* 207.1, 174.1, 172.2, 97.2 (Rha-C-1′), 74.4, 72.9, 68.7, 68.1, 66.0, 38.2, 34.3, 31.8, 31.2, 29.4, 29.3, 29.2, 28.2, 26.0, 24.6, 22.6, 22.3, 17.4, 14.1, 13.9; HRESIMS calcd for C_25_H_44_O_8_Na [M + Na]^+^ 495.2934; found, 495.2947.

#### 3.2.3. General Procedure for the Preparation of **25**–**30**

To a solution of compound **24** (354 mg, 1.0 mmol), **18**–**23** (637 mg for **18**, **19**, **21**; 508 mg for **20**; 566 mg for **22**; 711 mg for **23**, 1.3 mmol, respectively) and 4 Å molecular sieves in dry CH_2_Cl_2_ (20 mL) were added TMSOTf (22 μL, 0.10 mmol) at −78 °C under argon. After stirring at −78 °C for 0.5 h and then at 0 °C for 0.5 h, to the reaction mixture was added **15** (378 mg, 0.80 mmol) in dried CH_2_Cl_2_ (2 mL) under argon. After stirring at 0 °C for 0.5 h, N-iodosuccinimide (289 mg, 1.30 mmol) and silver trifluoromethanesulfonate (46 mg, 0.16 mmol) were added. The reaction was stirred for an additional 4 h while warming to room temperature. After the reaction was complete as judged by TLC, the reaction mixture was filtered and concentrated under reduced pressure. Then the residue was diluted with CH_2_Cl_2_ (100 mL), and washed with aqueous Na_2_S_2_O_3_ (50 mL), satd aq NaHCO_3_ (50 mL), and brine (2 × 50 mL). The organic layer was dried over Na_2_SO_4_, and concentrated in vacuo. The filtrate was concentrated and purified by silica gel column chromatography (1:2, EtOAc- petroleum ether) to furnish **25**–**30** as a white amorphous solid, respectively.

*1-Octyloxy-2,4-di-O-acetyl-3-O-levulinoylα-l-rhamnopyranosyl-(1→3)-2,4-di-O-acetyl-α-l-rhamnopyranosyl-(1→3)-4-O-hexanoyl-2-O-levulinoyl-α-l-rhamno-pyranoside* (**25**)*.* 86% for two steps; ^1^H-NMR (CDCl_3_): *δ* 5.29 (dd, 1H, *J* = 3.6, 1.7 Hz, H-2′′′), 5.27 (dd, 1H, *J* = 10.1, 3.5 Hz, H-3′′′), 5.19 (d, 1H, *J* = 2.3 Hz, H-1′), 5.10 (dd, 1H, *J* = 3.5, 1.7 Hz, H-2′′), 5.05 (t, 1H, *J* = 10.1 Hz, H-4′′′), 5.00 (t, 1H, *J* = 9.7 Hz, H-4′′), 4.90 (t, 1H, *J* = 10.0 Hz, H-4′), 4.85 (d, 1H, *J* = 1.4 Hz, H-1′′), 4.67 (dd, 1H, *J* = 4.3, 2.4 Hz, H-2′), 4.61 (d, 1H, *J* = 1.4 Hz, H-1′′′), 4.20–4.25 (m, 1H, H-5′′′), 4.07 (dd, 1H, *J* = 9.9, 3.6 Hz, H-3′′), 3.98 (dd, 1H, *J* = 9.8, 4.3 Hz, H-3′), 3.76–3.79 (m, 1H, H-5′′), 3.60–3.64 (m, 1H, H-5′), 3.43–3.48 (m, 1H, H-1-1), 3.35–3.39 (m, 1H, H-1-2), 2.77–2.87 (m, 2H, CH_2_CO), 2.73–2.76 (m, 1H, CH_2_CO-1), 2.60–2.65 (m, 2H, CH_2_CO), 2.52–2.57 (m, 2H, CH_2_CO), 2.40–2.45 (m, 1H, CH_2_CO-2), 2.31 (td, 2H, *J* = 7.1, 1.3 Hz, COCH_2_), 2.19, 2.17, 2.14, 2.12, 2.11, 1.70 (each s, each 3H, each CH_3_CO), 1.54–1.65 (m, 4H, 2 × CH_2_), 1.26–1.34 (m, 14H, 7 × CH_2_), 1.20 (d, 3H, *J* = 6.2 Hz, H-6′), 1.19 (d, 3H, *J* = 6.2 Hz, H-6), 1.16 (d, 3H, *J* = 6.2 Hz, H-6′′′), 0.92 (t, 3H, *J* = 7.0 Hz, CH_3_), 0.90 (t, 3H, *J* = 7.0 Hz, CH_3_); ^13^C-NMR (CDCl_3_): *δ* 206.3, 206.1, 172.3, 172.2, 171.2, 170.4, 170.2, 170.0, 99.8, 97.4, 97.2, 78.8, 77.8, 72.1, 71.3, 71.1, 70.5, 70.2, 69.5, 69.3, 69.1, 68.1, 67.2, 66.4, 37.7, 37.6, 34.3, 31.8, 31.3, 29.9, 29.7, 29.4, 29.3, 29.2, 28.0, 27.8, 26.1, 24.6, 22.6, 22.4, 22.3, 21.0, 20.8, 20.7, 17.6, 17.5, 16.7, 14.1, 13.9; HRESIMS calcd for C_50_H_78_O_22_Na [M + Na]^+^ 1053.4882; found, 1053.4885.

*1-Octyloxy-3,4-di-O-acetyl-2-O-levulinoyl-α-l-rhamnopyranosyl-(1→3)-2,4-di-O-acetyl-α-l-rhamnopyranosyl-(1→3)-4-O-hexanoyl-2-O-levulinoyl-α-l-rhamno-pyranoside* (**26**)*.* 88% for two steps; ^1^H-NMR (CDCl_3_): *δ* 5.10–5.14 (m, 3H, H-3′′′, H-2′′, H-2′′′), 5.06 (t, 1H, *J* = 10.1 Hz, H-4′′′), 5.04 (t, 1H, *J* = 10.0 Hz, H-4′′), 5.00 (t, 1H, *J* = 9.7 Hz, H-4′), 4.98 (dd, 1H, *J* = 3.4, 1.6 Hz, H-2′), 4.96 (d, 1H, *J* = 1.3 Hz, H-1′′′), 4.84 (d, 1H, *J* = 1.4 Hz, H-1′′), 4.66 (d, 1H, *J* = 1.5 Hz, H-1′), 4.11 (dd, 1H, *J* = 10.0, 3.4 Hz, H-3′′), 4.06 (dd, 1H, *J* = 9.7, 3.4 Hz, H-3′), 3.74–3.84 (m, 3H, H-5′, H-5′′, H-5′′′), 3.61–3.65 (m, 1H, H-1-1), 3.37–3.41 (m, 1H, H-1-2), 2.69–2.80 (m, 6H, 3 × COCH_2_), 2.66 (t, 2H, *J* = 6.5 Hz, COCH_2_), 2.42–2.48 (m, 1H, COCH_2_-1), 2.31–2.37 (m, 1H, COCH_2_-2), 2.22, 2.20, 2.17, 2.13, 2.05, 1.98 (each s, each 3H, each COCH_3_), 1.54–1.66 (m, 4H, 2 × CH_2_), 1.29–1.33 (m, 14H, 7 × CH_2_), 1.19 (d, 3H, *J* = 6.2 Hz, H-6′), 1.18 (d, 3H, *J* = 6.2 Hz, H-6′′), 1.17 (d, 3H, *J* = 6.2 Hz, H-6′′′), 0.90 (t, 3H, *J* = 7.2 Hz, CH_3_), 0.89 (t, 3H, *J* = 7.2 Hz, CH_3_); ^13^C-NMR (CDCl_3_): *δ* 206.0, 205.9, 173.0, 172.0, 171.5, 170.4, 170.2, 170.0, 169.8, 99.4, 98.6, 97.2, 75.2, 74.6, 72.3, 71.8, 71.4, 70.8, 70.0, 68.7, 68.1, 67.3, 67.0, 66.5, 37.8 (two), 34.0, 31.8, 31.3, 29.8, 29.7, 29.3 (two), 29.2, 28.2, 28.0, 26.1, 24.5, 22.6, 22.3, 20.9, 20.8, 20.6, 17.5, 17.2 (two), 14.1, 13.9; HRESIMS calcd for C_50_H_78_O_22_Na [M + Na]^+^ 1053.4882; found, 1053.4895.

*1-Octyloxy-2,3-O-isopropylidene-4-O-acetyl-α-l-rhamnopyranosyl-(1→3)-2,4-di-O-acetyl-α-l-rhamnopyranosyl-(1→3)-4-O-hexanoyl-2-O-levulinoyl-α-l-rhamno-pyranoside* (**27**)*.* 83% for two steps; ^1^H-NMR (CDCl_3_): *δ* 5.19 (s, 1H, H-1′′′), 5.13 (dd, 1H, *J* = 3.2, 1.7 Hz, H-2′′), 5.05 (t, 1H, *J* = 10.1 Hz, H-4′), 5.03 (t, 1H, *J* = 10.0 Hz, H-4′′), 4.93 (dd, 1H, *J* = 3.2, 1.5 Hz, H-2′), 4.82 (d, 1H, *J* = 1.2 Hz, H-1′′), 4.80 (dd, 1H, *J* = 10.1, 8.1 Hz, H-4′′′), 4.66 (d, 1H, *J* = 1.3 Hz, H-1′), 4.18 (dd, 1H, *J* = 10.0, 3.4 Hz, H-3′′′), 4.05–4.09 (m, 2H, H-3′, H-3′′′), 4.01 (d, 1H, *J* = 5.5 Hz, H-2′′′), 3.82–3.87 (m, 1H, H-5′′′), 3.75–3.77 (m, 1H, H-5′′), 3.61–3.65 (m, 2H, H-5′, H-1-1), 3.37–3.43 (m, 1H, H-1-2), 2.67–2.87 (m, 4H, 2 × COCH_2_), 2.30–2.44 (m, 2H, COCH_2_), 2.24, 2.14, 2.12, 2.09 (each s, each 3H, each CH_3_CO), 1.56–1.63 (m, 4H, 2 × CH_2_), 1.53 (s, 3H, CH_3_), 1.32 (s, 3H, CH_3_), 1.26–1.30 (m, 14H, 7 × CH_2_), 1.18 (d, 3H, *J* = 6.2 Hz, H-6′), 1.17 (d, 3H, *J* = 6.2 Hz, H-6′′), 1.10 (d, 3H, *J* = 6.2 Hz, H-6′′′), 0.90 (t, 3H, *J* = 7.0 Hz, CH_3_), 0.89 (t, 3H, *J* = 7.0 Hz, CH_3_); ^13^C-NMR (CDCl_3_): *δ* 206.1, 172.9, 171.9, 170.3, 170.2 (two), 170.0, 109.6, 99.3, 97.2, 76.1, 75.6, 75.0, 74.2, 74.1, 72.6, 72.2, 72.0, 71.8, 70.8, 68.2, 67.3, 66.5, 64.6, 37.8, 34.0, 31.8, 31.3, 29.7, 29.3 (two), 29.2, 28.3, 27.6, 26.3, 26.1, 24.5, 22.6, 22.3, 21.0 (two), 20.9, 17.4, 17.2, 16.5, 14.1, 13.9; HRESIMS calcd for C_49_H_78_O_20_Na [M + Na]^+^ 1009.4984; found, 1009.4988.

*1-Octyloxy-4-O-hexanoyl-2-O-levulinoyl-α-l-rhamnopyranosyl-(1→3)-2,4-di-O-acetyl-α-l-rhamnopyranosyl-(1→3)-2,3-di-O-acetyl-4-O-levulinoyl-α-l-rhamnopyranoside* (**28**)*.* 88% for two steps; ^1^H-NMR (CDCl_3_): *δ* 5.16 (dd, 1H, *J* = 10.2, 3.4 Hz, H-3′′′), 5.12 (dd, 1H, *J* = 3.3, 1.7 Hz, H-2′′′), 5.08 (dd, 1H, *J* = 3.4, 1.8 Hz, H-2′′), 5.04 (t, 1H, *J* = 9.9 Hz, H-4′′′), 5.03 (t, 1H, *J* = 9.9 Hz, H-4′′), 5.02 (t, 1H, *J* = 9.9 Hz, H-4′), 4.99 (dd, 1H, *J* = 3.2, 1.6 Hz, H-2′), 4.97 (d, 1H, *J* = 1.7 Hz, H-1′′′), 4.84 (d, 1H, *J* = 1.3 Hz, H-1′′), 4.64 (d, 1H, *J* = 1.5 Hz, H-1′), 4.10 (dd, 1H, *J* = 10.2 3.4 Hz, H-3′′), 4.05 (dd, 1H, *J* = 10.0, 3.4 Hz, H-3′), 3.74–3.84 (m, 3H, H-5′, H-5′′, H-5′′′), 3.61–3.65 (m, 1H, H-1-1), 3.36–3.40 (m, 1H, H-1-2), 2.68–2.78 (m, 6H, 3 × COCH_2_), 2.53 (td, 2H, *J* = 6.4, 2.2 Hz, COCH_2_), 2.42–2.47 (m, 1H, COCH_2_-1), 2.31–2.36 (m, 1H, COCH_2_-2), 2.21, 2.18, 2.16, 2.15, 2.11, 2.01 (each s, each 3H, each CH_3_CO), 1.55–1.64 (m, 4H, 2 × CH_2_), 1.26–1.33 (m, 14H, 7 × CH_2_), 1.18 (d, 3H, *J* = 6.2 Hz, H-6′′′), 1.17 (d, 3H, *J* = 6.2 Hz, H-6′′), 1.14 (d, 3H, *J* = 6.2 Hz, H-6′), 0.89 (t, 3H, *J* = 7.0 Hz, CH_3_), 0.89 (t, 3H, *J* = 7.2 Hz, CH_3_); ^13^C-NMR (CDCl_3_): *δ* 206.1, 206.0, 173.0, 171.9, 171.8, 170.4, 170.2, 170.0, 169.9, 99.4, 98.6, 97.2, 75.2, 74.7, 72.2, 71.9, 71.8, 71.4, 71.0, 70.1, 68.4, 68.2 (two), 67.3, 67.1, 66.6, 37.6, 34.0, 31.8, 31.3, 29.7 (two), 29.3 (two), 28.2, 27.9, 26.1, 24.5, 22.6, 22.3, 20.9 (two), 20.8, 20.7, 17.5, 17.2, 17.1, 14.1, 13.9; HRESIMS calcd for C_50_H_78_O_22_Na [M + Na]^+^ 1053.4882; found, 1053.4893.

*1-Octyloxy-2,3,4-tri-O-acetyl-α-l-rhamnopyranosyl-(1→3)-2,4-di-O-acetyl-α-l-rhamnopyranosyl-(1→3)-4-O-hexanoyl-2-O-levulinoyl-α-l-rhamnopyranoside* (**29**)*.* 89% for two steps; ^1^H-NMR (CDCl_3_): *δ* 5.15 (dd, 1H, *J* = 10.2, 3.4 Hz, H-3′′′), 5.13 (dd, 1H, *J* = 3.2, 1.6 Hz, H-2′′′), 5.10 (dd, 1H, *J* = 3.2, 1.8 Hz, H-2′′), 5.07 (t, 1H, *J* = 9.8 Hz, H-4′′′), 5.01–5.06 (m, 2H, H-4′, H-4′′), 4.98–4.99 (m 2H, H-2′, H-1′′′), 4.84 (d, 1H, *J* = 1.7 Hz, H-1′′), 4.65 (d, 1H, *J* = 1.4 Hz, H-1′), 4.11 (dd, 1H, *J* = 10.0, 3.4 Hz, H-3′′), 4.06 (dd, 1H, *J* = 10.0, 3.1 Hz, H-3′), 3.75–3.78 (m, 3H, H-5′, H-5′′, H-5′′′), 3.61–3.65 (m, 1H, H-1-1), 3.37–3.41 (m, 1H, H-1-2), 2.77 (t, 2H, *J* = 6.1 Hz, COCH_2_), 2.66–2.72 (m, 2H, CH_2_CO), 2.30–2.78 (m, 2H, CH_2_CO), 2.22, 2.17, 2.15, 2.13, 2.05, 1.97 (each s, each 3H, each CH_3_CO), 1.55–1.66 (m, 4H, 2 × CH_2_), 1.28–1.33 (m, 14H, 7 × CH_2_), 1.18 (d, 3H, *J* = 6.2 Hz, H-6′), 1.17 (d, 6H, *J* = 6.2 Hz, H-6′′, H-6′′′), 0.90 (t, 6H, *J* = 7.0 Hz, 2 × CH_3_); ^13^C-NMR (CDCl_3_): *δ* 206.0, 173.0, 171.9, 170.4, 170.2, 170.1, 170.0, 169.7, 99.4, 98.7, 97.2, 75.2, 74.8, 72.3, 71.9, 71.8, 71.4, 70.9, 70.0, 68.7, 68.2, 67.3, 67.1, 66.5, 37.7, 34.0, 31.8, 31.3, 29.7 (two), 29.3 (two), 29.2, 28.2, 26.1, 24.5, 22.6, 22.3, 20.9 (two), 20.8, 20.7, 17.5, 17.2 (two), 14.1, 13.9; HRESIMS calcd for C_47_H_74_O_21_Na [M + Na]^+^ 997.4620; found, 997.4630.

*1-Octyloxy-2-O-acetyl-3,4-di-O-levulinoyl-α-l-rhamnopyranosyl-(1→3)-2,4-di-O-acetyl-α-l-rhamnopyranosyl-(1→3)-4-O-hexanoyl-2-O-levulinoyl-α-l-rhamno-pyranoside* (**30**)*.* 84% for two steps; ^1^H-NMR (CDCl_3_): *δ* 5.16 (dd, 1H, *J* = 10.3, 3.4 Hz, H-3′′′), 5.12 (dd, 1H, *J* = 3.3, 1.7 Hz, H-2′′′), 5.06 (t, 1H, *J* = 9.8 Hz, H-4′′′), 5.05 (dd, 1H, *J* = 3.4, 1.7 Hz, H-2′′), 5.04 (t, 1H, *J* = 9.9 Hz, H-4′′), 5.03 (t, 1H, *J* = 9.9 Hz, H-4′), 4.98 (dd, 1H, *J* = 3.4, 1.6 Hz, H-2′), 4.47 (d, 1H, *J* = 1.7 Hz, H-1′′′), 4.83 (d, 1H, *J* = 1.4 Hz, H-1′′), 4.65 (d, 1H, *J* = 1.6 Hz, H-1′), 4.10 (dd, 1H, *J* = 10.1, 3.4 Hz, H-3′′), 4.05 (dd, 1H, *J* = 10.1, 3.4 Hz, H-3′), 3.73–3.83 (m, 3H, H-5′, H-5′′, H-5′′′), 3.61–3.65 (m, 1H, H-1-1), 3.37–3.40 (m, 1H, H-1-2), 2.31–2.78 (m, 14H, 7 × COCH_2_), 2.21, 2.18, 2.16, 2.16, 2.14, 2.12 (each s, each 3H, each COCH_3_), 1.55–1.65 (m, 4H, 2 × CH_2_), 1.25–1.33 (m, 14H, 7 × CH_2_), 1.18 (d, 3H, *J* = 6.2 Hz, H-6′), 1.17 (d, 3H, *J* = 6.2 Hz, H-6′′), 1.16 (d, 3H, *J* = 6.2 Hz, H-6′′′), 0.90 (t, 3H, *J* = 7.0 Hz, CH_3_), 0.89 (t, 3H, *J* = 7.0 Hz, CH_3_); ^13^C-NMR (CDCl_3_): *δ* 206.4 (two), 173.0, 172.0, 171.9, 171.4, 170.4, 170.2, 170.0, 99.4, 98.7, 97.2, 75.2, 74.8, 72.2, 71.9, 71.8, 71.4, 70.8, 70.0, 68.7, 68.1, 67.3, 67.1, 66.5, 37.8, 37.7 (two), 34.0, 31.8, 31.3, 29.7, 29.3 (two), 29.2, 28.2, 27.9, 27.8, 26.1, 24.5, 22.6, 22.3, 20.9 (two), 20.8, 17.5, 17.2, 17.1, 14.1, 13.9; HRESIMS calcd for C_53_H_82_O_23_Na [M + Na]^+^ 1109.5145; found, 1109.5156.

#### 3.2.4. General Method A for Removal of the Lev Protecting Groups

To a stirred solution of Lev protected compound (1.0 mmol) in 20 mL dry CH_2_Cl_2_-MeOH (*v*:*v* = 1:1) hydrazine acetate (10 mmol)was added. After stirring at room temperature for 5 h, the reaction mixture was concentrated. The residue was purified by silica gel column chromatography (1:1, petroleum ether–EtOAc) to afford a white solid **2**, **3**, and **5**–**7**, respectively.

*1-Octyloxy-2,4-di-O-acetyl-α-l-rhamnopyranosyl-(1→3)-2,4-di-O-acetyl-α-l-rhamnopyranosyl-(1→3)-4-O-hexanoyl-α-l-rhamnopyranoside* (**2**)*.* **2** was prepared from **25** (515 mg, 0.50 mmol) and hydrazine acetate (921 mg, 10 mmol) following the general method A in 88% yield. ^1^H-NMR (CDCl_3_): *δ* 5.41 (d, 1H, *J* = 2.3 Hz, H-1′′′), 4.99 (t, 1H, *J* = 9.8 Hz, H-4′), 4.97(t, 1H, *J* = 9.8 Hz, H-4′′), 4.95 (s, 1H, H-1′′), 4.91 (dd, 1H, *J* = 3.5, 1.8 Hz, H-2′′), 4.86 (t, 1H, *J* = 9.9 Hz, H-4′′′), 4.74 (d, 1H, *J* = 1.7 Hz, H-1′), 4.44 (dd, 1H, *J* = 4.0, 2.5 Hz, H-2′′′), 4.05–4.08 (m, 2H, H-3′, H-3′′), 3.96–3.98 (m, 1H, H-5′′), 3.93 (dd, 1H, *J* = 3.1, 1.7 Hz, H-2′), 3.83 (dd, 1H, *J* = 9.8, 4.1 Hz, H-3′′′), 3.76–3.79 (m, 1H, H-5′′′), 3.63–3.67 (m, 1H, H-1-1), 3.40–3.44 (m, 1H, H-5′), 3.37–3.39 (m, 1H, H-1-2), 2.32 (t, 2H, *J* = 6.4 Hz, COCH_2_), 2.17, 2.16, 2.15, 1.73 (each s, each 3H, each CH_3_CO), 1.61–1.66 (m, 2H, CH_2_), 1.55–1.59 (m, 2H, CH_2_), 1.26–1.33 (m, 16H, CH_2_), 1.21 (d, 3H, *J* = 6.1 Hz, H-6′′′), 1.20 (d, 3H, *J* = 6.2 Hz, H-6′), 1.18 (d, 3H, *J* = 6.3 Hz, H-6′′), 0.90 (t, 6H, *J* = 7.0 Hz, 2 × CH_3_); ^13^C-NMR (CDCl_3_): *δ* 172.6, 171.6, 170.5, 170.2, 124.3, 99.4, 98.8, 97.4, 78.3, 77.5, 74.3, 72.8, 71.3 (two), 71.2, 70.8, 69.2, 67.8, 67.7, 66.9, 66.2, 34.3, 31.8, 31.3, 29.4, 29.3, 29.2, 26.1, 25.2, 24.6, 22.6, 22.3, 21.0 (two), 20.7, 17.5, 17.4, 17.3, 14.1, 13.9; HRESIMS calcd for C_40_H_66_O_18_Na [M + Na]^+^ 857.4147; found, 857.4160.

*1-Octyloxy-3,4-di-O-acetyl-α-L-rhamnopyranosyl-(1→3)-2,4-di-O-acetyl-α-l-rhamnopyranosyl-(1→3)-4-O-hexanoyl-α-l-rhamnopyranoside* (**3**)*.* Analogously, **3** was prepared from **26** (773 mg, 0.75 mmol) and hydrazine acetate (1.38 g, 15 mmol) following the general method A in 85% yield. ^1^H-NMR (CDCl_3_): *δ* 5.08 (t, 1H, *J* = 10.0 Hz, H-4′), 5.06 (t, 1H, *J* = 10.0 Hz, H-4′′′), 5.05 (t, 1H, *J* = 10.0 Hz, H-4′′), 5.03 (dd, 1H, *J* = 9.9, 3.3 Hz, H-3′′′), 5.02 (dd, 1H, *J* = 3.3, 1.7 Hz, H-2′′), 4.97 (d, 1H, *J* = 1.6 Hz, H-1′′′), 4.88(d, 1H, *J* = 1.4 Hz, H-1′′), 4.77 (d, 1H, *J* = 1.4 Hz, H-1′), 4.15 (dd, 1H, *J* = 9.9, 3.4 Hz, H-3′′), 3.98 (dd, 1H, *J* = 3.3, 1.7 Hz, H-2′′′), 3.95–3.97 (m, 1H, H-5′′), 3.95 (dd, 1H, *J* = 3.2, 1.8 Hz, H-2′), 3.92 (dd, 1H, *J* = 9.8, 3.3 Hz, H-3′), 3.77–3.83 (m, 2H, H-5′, H-5′′′), 3.67 (dt, 1H, *J* = 9.5, 6.5 Hz, H-1-1), 3.41 (dt, 1H, *J* = 9.6, 6.7 Hz, H-1-2), 2.45 (dt, 1H, *J* = 15.7, 7.4 Hz, COCH_2_-1), 2.34 (dt, 1H, *J* = 15.7, 7.9 Hz, COCH_2_-2), 2.18, 2.10, 2.07, 2.04 (each s, each 3H, each CH_3_CO), 1.63–1.66 (m, 2H, CH_2_), 1.56–1.59 (m, 2H, CH_2_), 1.29–1.33 (m, 14H, 7 × CH_2_), 1.21 (d, 3H, *J* = 6.2 Hz, H-6′′′), 1.19 (d, 3H, *J* = 6.2 Hz, H-6′′), 1.18 (d, 3H, *J* = 6.3 Hz, H-6′), 0.90 (t, 3H, *J* = 7.2 Hz, CH_3_), 0.89 (t, 3H, *J* = 7.2 Hz, CH_3_); ^13^C-NMR (CDCl_3_): *δ* 173.4, 170.3 (two), 170.0, 169.9, 101.0, 99.3 (two), 78.9, 74.7, 72.3, 71.8, 71.5, 71.4, 71.1 (two), 69.5, 68.0, 67.2, 67.1, 66.2, 34.1, 31.8, 31.3, 29.4, 29.3, 29.2, 26.1, 24.5, 22.6, 22.3, 21.0, 20.9, 20.8 (two), 17.4, 17.2, 14.1, 13.9; HRESIMS calcd for C_40_H_66_O_18_Na [M + Na]^+^ 857.4147; found, 857.4161.

*1-Octyloxy-2,3-di-O-acetyl-α-l-rhamnopyranosyl-(1→3)-2,4-di-O-acetyl-α-l-rhamnopyranosyl-(1→3)-4-O-hexanoyl-α-l-rhamnopyranoside* (**5**)*.* Analogously, **5** was prepared from **28** (515 mg, 0.50 mmol) and hydrazine acetate (921 mg, 10 mmol) following the general method A in 87% yield. ^1^H-NMR (CDCl_3_): *δ* 5.07 (t, 1H, *J* = 9.7 Hz, H-4′), 5.06 (dd, 1H, *J* = 9.7, 3.3 Hz, H-3′′), 5.05 (t, 1H, *J* = 9.7 Hz, H-4′′), 4.98–5.01 (m, 2H, H-2′′, H-2′′′), 4.89 (d, 1H, *J* = 1.7 Hz, H-1′′′), 4.87 (d, 1H, *J* = 1.4 Hz, H-1′′), 4.77 (d, 1H, *J* = 1.4 Hz, H-1′), 4.10 (dd, 1H, *J* = 10.0, 3.5 Hz, H-3′′), 3.95 (dd, 1H, *J* = 3.3, 1.2 Hz, H-2′), 3.91–3.94 (m, 2H, H-3′, H-5′′′), 3.73–3.80 (m, 2H, H-5′, H-5′′), 3.66 (dt, 1H, *J* = 9.6, 6.7 Hz, H-1-1), 3.56 (t, 1H, *J* = 9.7 Hz, H-4′′′), 3.40 (dt, 1H, *J* = 9.6, 6.5 Hz, H-1-2), 2.42 (dt, 1H, *J* = 15.8, 7.5 Hz, COCH_2_-1), 2.34 (dt, 1H, *J* = 15.7, 7.8 Hz, COCH_2_-2), 2.18, 2.13, 2.12, 2.08 (each s, each 3H, each CH_3_CO), 1.62–1.66 (m, 2H, CH_2_), 1.57–1.59 (m, 2H, CH_2_), 1.33 (d, 3H, *J* = 6.2 Hz, H-6′′′), 1.28–1.31 (m, 14H, 7 × CH_2_), 1.21 (d, 3H, *J* = 6.2 Hz, H-6′′), 1.19 (d, 3H, *J* = 6.2 Hz, H-6′), 0.90 (t, 3H, *J* = 7.1 Hz, CH_3_), 0.89 (t, 3H, *J* = 7.2 Hz, CH_3_); ^13^C-NMR (CDCl_3_): *δ* 173.0, 171.1, 170.5, 170.2, 170.0, 99.3, 99.2, 98.6, 78.8, 74.0, 72.2, 71.7, 71.3 (two), 71.0, 70.4, 69.6, 68.0, 67.3, 66.2, 34.1, 31.8, 31.3, 29.4, 29.3, 29.2, 26.1, 24.5, 22.6, 22.3, 20.9 (two), 20.8, 20.7, 17.4 (three), 14.1, 13.9; HRESIMS calcd for C_40_H_66_O_18_Na [M + Na]^+^ 857.4147; found, 857.4160.

*1-Octyloxy-2,3,4-tri-O-acetyl-α-l-rhamnopyranosyl-(1→3)-2,4-di-O-acetyl-α-l-rhamnopyranosyl-(1→3)-4-O-hexanoyl-α-l-rhamnopyranoside* (**6**)*.* Analogously, **6** was prepared from **29** (488 mg, 0.50 mmol) and hydrazine acetate (461 mg, 5 mmol) following the general method A in 88% yield. ^1^H-NMR (CDCl_3_): *δ* 5.16 (dd, 1H, *J* = 10.2, 3.3 Hz, H-3′′′), 5.09 (t, 1H, *J* = 9.9 Hz, H-4′), 5.02–5.06 (m, 4H, H-4′′, H-4′′′, H-2′′′, H-2′′), 4.89, 4.88, 4.77 (each brs, each 1H, H-1′, H-1′′, H-1′′′), 4.08 (dd, 1H, *J* = 9.9, 3.4 Hz, H-3′′), 3.95 (dd, 1H, *J* = 3.3, 1.2 Hz, H-2′), 3.92 (dd, 1H, *J* = 9.9, 3.3 Hz, H-3′), 3.91–3.93 (m, 1H, H-5′′′), 3.82–3.84 (m, 1H, H-5′′), 3.76–3.78 (m, 1H, H-5′), 3.66 (dt, 1H, *J* = 9.5, 2.8 Hz, H-1-1), 3.40 (dt, 1H, *J* = 9.6, 6.6 Hz, H-1-2), 2.44 (dt, 1H, *J* = 16.0, 7.5 Hz, COCH_2_-1), 2.34 (dt, 1H, *J* = 16.0, 7.7 Hz, COCH_2_-2), 2.18, 2.14, 2.13, 2.05, 1.98 (each s, each 3H, each CH_3_CO), 1.62–1.66 (m, 2H, CH_2_), 1.55–1.58 (m, 2H, CH_2_), 1.26–1.33 (m, 14H, 7 × CH_2_), 1.21 (d, 3H, *J* = 6.4 Hz, H-6′′′), 1.19 (d, 3H, *J* = 6.4 Hz, H-6′′), 1.18 (d, 3H, *J* = 6.3 Hz, H-6′), 0.90 (t, 3H, *J* = 7.1 Hz, CH_3_), 0.89 (t, 3H, *J* = 7.2 Hz, CH_3_); ^13^C-NMR (CDCl_3_): *δ* 173.0, 170.3, 170.2, 170.1, 170.0, 169.7, 99.2 (two), 98.8, 78.7, 74.8, 72.0, 71.7, 71.3, 71.0, 70.8, 68.6, 68.0, 67.4, 67.2, 66.2, 34.1, 31.8, 31.3, 29.4, 29.3, 29.2, 26.1, 24.5, 22.6, 22.3, 20.9 (two), 20.8, 20.7 (two), 17.4, 17.2, 14.1, 13.9; HRESIMS calcd for C_42_H_68_O_19_Na [M + Na]^+^ 899.4252; found, 899.4263.

*1-Octyloxy-2-O-acetyl-α-l-rhamnopyranosyl-(1→3)-2,4-di-O-acetyl-α-L-rhamnopyranosyl-(1→3)-4-O-hexanoyl-α-l-rhamnopyranoside* (**7**)*.* Analogously, **7** was prepared from **30** (544 mg, 0.50 mmol) and hydrazine acetate (1.38 g, 15 mmol) following the general method A in 78% yield. ^1^H-NMR (CDCl_3_): *δ* 5.05 (t, 1H, *J* = 9.7 Hz, 1H, H-4′), 5.04 (t, 1H, *J* = 9.7 Hz, H-4′′), 4.98 (dd, 1H, *J* = 3.4, 1.8 Hz, H-2′′), 4.89 (dd, 1H, *J* = 3.6, 1.5 Hz, H-2′′′), 4.87 (d, 1H, *J* = 1.8 Hz, H-1′′′), 4.87 (d, 1H, *J* = 1.6 Hz, H-1′′), 4.75 (d, 1H, *J* = 1.8 Hz, H-1′), 4.04 (dd, 1H, *J* = 9.7, 3.4 Hz, H-3′′), 3.94 (dd, 1H, *J* = 9.9, 3.3 Hz, H-3′), 3.93 (dd, 1H, *J* = 3.3, 1.5 Hz, H-2′), 3.92 (dq, 1H, *J* = 9.7, 6.3 Hz, H-5′), 3.78 (dd, 1H, *J* = 9.4, 3.5 Hz, H-3′′′), 3.76 (dq, 1H, *J* = 9.7, 6.3 Hz, H-5′′), 3.64–3.67 (m, 1H, H-1-1), 3.58–3.62 (m, 1H, H-5′′′), 3.43 (t, 1H, *J* = 9.6 Hz, H-4′′′), 3.40 (ddd, 1H, *J* = 10.4, 6.8, 6.8 Hz, H-1-2), 2.40–2.44 (m, 1H, COCH_2_-1), 2.34–2.37 (m, 1H, COCH_2_-2), 2.14, 2.13, 2.12 (each s, each 3H, each COCH_3_), 1.61–1.63 (m, 4H, 2 × CH_2_), 1.27–1.33 (m, 14H, 7 × CH_2_), 1.21 (d, 3H, *J* = 6.4 Hz, H-6′′′), 1.19 (d, 6H, *J* = 6.4 Hz, H-6′′, H-6′), 0.89 (t, 6H, *J* = 7.1 Hz, 2 × CH_3_); ^13^C-NMR (CDCl_3_): *δ* 173.0, 170.5, 170.4, 170.2, 99.3, 99.2, 99.2, 78.7, 74.7, 73.3, 72.5, 72.3, 71.9, 71.8, 71.0, 69.8, 69.1, 68.2, 67.3, 66.2, 34.1, 31.8, 31.3, 29.4, 29.3, 29.2, 28.1, 24.5, 22.6, 22.3, 21.0, 20.9, 20.8, 17.4, 17.2, 17.4, 14.1, 14.9; HRESIMS calcd for C_38_H_64_O_17_Na [M + Na]^+^ 815.4041; found, 815.4058.

#### 3.2.5. 1-Octyloxy-4-O-acetyl-α-l-rhamnopyranosyl-(1→3)-2,4-di-O-acetyl-α-l-rhamnopyranosyl-(1→3)-4-*O*-hexanoyl-α-l-rhamnopyranoside (**4**)

To a solution of compound **30** (400 mg, 0.43 mmol) in 10 mL dry CH_2_Cl_2_-MeOH (*V*:*V* = 1:1) hydrazine acetate was added (396 mg, 4.3 mmol) and stirred at room temperature for 5 h under argon. Then the reaction mixture was concentrated under reduced pressure, and then the mixture was diluted with EtOAc (100 mL), washed with saturated aqueous NaHCO_3_ (2 × 50 mL), 1 mol·L^−1^ HCl (2 × 50 mL), and brine (2 × 50 mL), dried over Na_2_SO_4_, and concentrated in vacuo. The above residue was dissolved in 80% HOAc (20 mL) and stirred for 2 h at 100 °C, then the reaction mixture was concentrated in vacuo. The residue was purified by silica gel column chromatography (3:1, CH_2_Cl_2_—EtOAc) to afford a white solid **4** (265 mg, 78% for two steps); ^1^H-NMR (CDCl_3_): *δ* 5.07 (t, 1H, *J* = 10.0 Hz, H-4′), 5.04 (brs, 1H, H-1′′′) 5.03 (t, 1H, *J* = 10.0 Hz, H-4′′), 4.98 (dd, 1H, *J* = 3.3, 1.9 Hz, H-2′′), 4.86 (d, 1H, *J* = 1.4 Hz, H-1′′), 4.80 (d, 1H, *J* = 1.7 Hz, H-1′), 4.79 (t, 1H, *J* = 10.0 Hz, H-4′′′), 4.18 (dd, 1H, *J* = 10.0, 3.4 Hz, H-3′′), 3.98 (dd, 1H, *J* = 9.9, 3.2 Hz, H-3′), 3.97 (dd, 1H, *J* = 3.2, 1.3 Hz, H-2′), 3.90 (dd, 1H, *J* = 9.9, 3.3 Hz, H-3′′′), 3.86 (dd, 1H, *J* = 3.2, 1.9 Hz, H-2′′′), 3.70–3.81 (m, 3H, H-5′, H-5′′, H-5′′′), 3.65 (dt, 1H, *J* = 9.5, 2.8 Hz, H-1-1), 3.41 (dt, 1H, *J* = 9.6, 6.7 Hz, H-1-2), 3.61, 3.19, 3.05 (each brs, each 1H, each OH), 2.46–2.50 (m,1 H, COCH_2_-1), 2.34–2.40 (m, 1H, COCH_2_-2), 2.15, 2.13, 2.08 (each s, each 3H, each CH_3_CO), 1.61–1.66 (m, 2H, CH_2_), 1.55–1.58 (m, 2H, CH_2_), 1.26–1.33 (m, 14H, 7 × CH_2_), 1.20, 1.18, 1.15 (each d, each 3H, *J* = 6.4 Hz, H-6′, H-6′′, H-6′′′), 0.90 (t, 3H, *J* = 7.2 Hz, CH_3_), 0.89 (t, 3H, *J* = 7.2 Hz, CH_3_); ^13^C-NMR (CDCl_3_): *δ* 173.6, 172.1, 170.2, 170.0, 101.5, 99.4, 99.3, 79.1, 75.1, 75.0, 72.3, 71.9, 71.8, 71.0, 70.9, 69.8, 68.0, 67.2, 66.5, 66.2, 34.1, 31.8, 31.3, 29.4, 29.3, 29.2, 26.1, 24.5, 22.6, 22.3, 21.1, 21.0, 20.8, 17.4 (two), 17.1, 14.1, 13.9; HRESIMS calcd for C_38_H_64_O_17_Na [M + Na]^+^ 815.4041; found, 815.4055.

#### 3.2.6. 1-Octyloxy-α-L-rhamnopyranosyl-(1→3)-2,4-di-*O*-acetyl-α-l-rhamnopyranosyl-(1→3)-4-*O*-hexanoyl-α-l-rhamnopyranoside (**8**)

To a solution of compounds **30** (543 mg, 0.50 mmol) in 20 mL dry CH_2_Cl_2_–MeOH (*V*:*V* = 1:1), hydrazine acetate (1.38 g, 15 mmol) was added and stirred at 40 °C for 18 h under argon. The reaction mixture was concentrated in vacuo, and then the residue was purified by silica gel column chromatography (2:1, CH_2_Cl_2_–EtOAc) to afford a white solid **8** (0.49 g, 71%). ^1^H-NMR (CDCl_3_): *δ* 5.06 (t, 1H, *J* = 9.9 Hz, H-4′), 5.03 (t, 1H, *J* = 10.0 Hz, H-4′′), 4.97 (dd, 1H, *J* = 3.2, 1.1 Hz, H-2′′), 4.96 (brs, 1H, H-1′′), 4.87 (brs, 1H, H-1′′′), 4.77 (brs, 1H, H-1′), 4.18 (dd, 1H, *J* = 9.9, 3.3 Hz, H-3′′), 3.95–3.99 (m, 1H, H-5′′), 3.95 (dd, 1H, *J* = 3.2, 1.5 Hz, H-2′), 3.91 (dd, 1H, *J* = 9.7, 3.1 Hz, H-3′), 3.82 (dd, 1H, *J* = 3.3, 1.2 Hz, H-2′′′), 3.76–3.79 (m, 1H, H-5′), 3.63–3.67 (m, 2H, H-3′′′, H-1-1), 3.55–3.58 (m, 1H, H-5′′′), 3.39–3.46 (m, 2H, H-4′′′, H-1-2), 2.49 (dt, 1H, *J* = 15.7, 7.5 Hz, COCH_2_-1), 2.36 (dt, 1H, *J* = 15.7, 7.9 Hz, COCH_2_-2), 2.14, 2.11 (each s, each 3H, each CH_3_CO), 1.62–1.66 (m, 2H, CH_2_), 1.55–1.58 (m, 2H, CH_2_), 1.28–1.31 (m, 14H, 7 × CH_2_), 1.26 (d, 3H, *J* = 6.2 Hz, H-6′′′), 1.19 (d, 3H, *J* = 6.2 Hz, H-6′′), 1.18 (d, 3H, *J* = 6.2 Hz, H-6′), 0.90 (t, 3H, *J* = 7.2 Hz, CH_3_), 0.89 (t, 3H, *J* = 7.2 Hz, CH_3_); ^13^C-NMR (CDCl_3_): *δ* 174.0, 170.4 (two), 101.9, 99.3 (two), 78.6, 75.0, 72.7, 72.2, 71.9, 71.3, 71.1, 70.9, 69.1, 68.0, 67.1, 66.2, 34.1, 31.8, 31.3, 29.4, 29.3, 29.2, 26.1, 24.5, 22.6, 22.2, 20.9 (two), 17.4 (two), 17.2, 14.1, 13.9; HRESIMS calcd for C_36_H_62_O_16_Na [M + Na]^+^ 773.3936; found, 773.3942.

#### 3.2.7. 1-Octyloxy-4-*O*-hexanoyl-2-*O*-acetyl-α-l-rhamnopyranoside (**32**)

To a solution of the known compound **31** (2.0 g, 5.4 mmol) in anhydrous *N,N*-dimethylformamide (20 mL), and triethylorthoacetate (1.5 mL, 7.9 mmol) were added, followed by a catalytic amount of camphorsulfonicacid (250 mg, 1.1 mmol). The mixture was stirred for 5 h at r.t. When TLC (1:1, petroleum ether-EtOAc) showed complete conversion, the mixture was diluted with EtOAc (200 mL). The organic layer was shaken with 1 mol·L^−1^ HCl (3 × 100 mL), followed by washing with water (3 × 100 mL), and brine (2 × 100 mL), dried over Na_2_SO_4_, and concentrated in vacuo. The residue was purified by silica gel column chromatography (8:1, petroleum ether—EtOAc) to generate **32** (1.9 g, 86%). ^1^H-NMR (CDCl_3_): *δ* 5.05 (dd, 1H, *J* = 3.6, 1.6 Hz, H-2′), 4.86 (t, 1H, *J* = 9.8 Hz, H-4′), 4.77 (d, 1H, *J* = 1.3 Hz, H-1′), 4.03 (dd, 1H, *J* = 9.9, 3.6 Hz, H-3′), 3.79–3.83 (m, 1H, H-5′), 3.66 (dt, 1H, *J* = 9.9, 6.8 Hz, H-1-1), 3.40 (dt, 1H, *J* = 9.9, 6.5 Hz, H-1-2), 2.36–2.39 (m, 2H, COCH_2_), 2.16 (s, 3H, CH_3_CO), 1.63–1.68 (m, 2H, H-2), 1.56–1.60 (m, 2H, H-3), 1.26–1.35 (m, 14H, 7 × CH_2_), 1.21 (d, 3H, *J* = 6.3 Hz, H-6′), 0.90 (t, 3H, *J* = 7.1 Hz, CH_3_); ^13^C-NMR (CDCl_3_) *δ* 174.4, 170.6, 97.1 (C-1′), 74.5, 72.9, 68.6, 68.2, 65.8, 34.3, 31.8, 31.2, 29.4, 29.3, 29.2, 26.1, 24.6, 22.6, 22.3, 21.0, 17.4, 14.1, 13.9; HRESIMS calcd for C_22_H_40_O_7_Na [M + Na]^+^ 439.2672; found, 439.2688.

#### 3.2.8. 1-Octyloxy-2,4-di-*O*-acetyl-3-*O*-levulinoyl-α-l-rhamnopyranosyl-(1→3)-2,4-di-*O*-acetyl-α-l-rhamnopyranosyl-(1→3)-4-*O*-hexanoyl-2-*O*-acetyl-α-l-rhamnopyranoside (**33**)

Analogously, **33** was prepared from **32**, **24** and **18** as described for **25** in 82% yield. ^1^H-NMR (CDCl_3_): *δ* 5.14 (dd, 1H, *J* = 10.2, 3.4 Hz, H-3′′′), 5.13 (dd, 1H, *J* = 3.6, 1.7 Hz, H-2′′′), 5.07 (t, 2H, *J* = 9.8 Hz, H-4′′, H-4′′′), 5.04 (t, 1H, *J* = 9.7 Hz, H-4′), 5.03 (dd, 1H, *J* = 3.4, 1.6 Hz, H-2′′), 4.97 (dd, 1H, *J* = 3.4, 1.7 Hz, H-2′), 4.85 (d, 1H, *J* = 1.5 Hz, H-1′′′), 4.84 (d, 1H, *J* = 1.6 Hz, H-1′′), 4.69 (d, 1H, *J* = 1.5 Hz, H-1′), 3.74–3.83 (m, 3H, H-5′, H-5′′, H-5′′′), 3.61–3.65 (m, 1H, H-1-1), 3.37–3.41 (m, 1H, H-1-2), 2.73–2.77 (m, 1H, COCH_2_-1), 2.60–2.66 (m, 1H, COCH_2_-2), 2.51–2.56 (m, 1H, COCH_2_-1′), 2.39–2.45 (m, 1H, COCH_2_), 2.30–2.36 (m, 1H, COCH_2_-2′), 2.18, 2.16, 2.14, 2.12, 2.09 (each s, each 3H, each CH_3_CO), 1.55–1.65 (m, 4H, 2 × CH_2_), 1.22–1.32 (m, 14H, 7 × CH_2_), 1.19 (d, 3H, *J* = 6.2 Hz, H-6′′′), 1.17 (d, 6H, *J* = 6.2 Hz, H-6′′, H-6′), 0.90 (t, 3H, *J* = 7.1 Hz, CH_3_), 0.89 (t, 3H, *J* = 7.2 Hz, CH_3_); ^13^C-NMR (CDCl_3_): *δ* 206.1, 172.9, 171.2, 170.5, 170.3 (two), 170.2, 170.1, 99.2, 98.8, 97.2, 75.5, 74.7, 72.2, 72.0, 71.8, 71.4, 70.5, 70.0, 68.7, 68.2, 67.4, 67.1, 66.5, 37.6, 34.0, 31.8, 31.3, 29.7, 29.3 (two), 29.2, 27.8, 26.1, 24.5, 22.6, 22.3, 22.1, 20.9 (two), 20.8 (two), 17.4 (two), 14.1, 13.9; HRESIMS calcd for C_47_H_74_O_21_Na [M + Na]^+^ 997.4620; found, 997.4633.

#### 3.2.9. 1-Octyloxy-2,4-di-*O*-acetyl-α-L-rhamnopyranosyl-(1→3)-2,4-di-*O*-acetyl-α-l-rhamnopyranosyl-(1→3)-4-*O*-hexanoyl-2-*O*-acetyl-α-l-rhamnopyranoside (**9**)

Analogously, **9** was prepared from **33** as described for **2** in 90% yield. ^1^H-NMR (CDCl_3_): *δ* 5.12 (dd, 1H, *J* = 3.4, 1.6 Hz, H-2′′′), 5.07 (t, 1H, *J* = 9.8 Hz, H-4′′′), 5.03 (t, 1H, *J* = 10.0 Hz, H-4′′), 4.93 (dd, 1H, *J* = 3.1, 1.8 Hz, H-2′′), 4.89 (s, 1H, H-1′′′), 4.87 (dd, 1H, *J* = 3.5, 1.4 Hz, H-2′), 4.83 (d, 1H, *J* = 1.1 Hz, H-1′′), 4.81 (t, 1H, *J* = 9.8 Hz, H-4′), 4.69 (s, 1H, H-1′), 4.06 (dd, 1H, *J* = 9.9, 3.3 Hz, H-3′′), 3.93 (dd, 1H, *J* = 10.0, 3.4 Hz, H-3′), 3.85 (dd, 1H, *J* = 10.0, 3.4 Hz, H-3′′′), 3.71–3.78 (m, 3H, H-5′, H-5′′, H-5′′′), 3.61–3.65 (m, 1H, H-1-1), 3.37–3.41 (m, 1H, H-1-2), 2.38–2.44 (m, 1H, COCH_2_-1), 2.30–2.35 (m, 1H, COCH_2_-2), 2.19, 2.15, 2.15, 2.14, 2.13 (each s, each 3H, each CH_3_CO), 1.54–1.65 (m, 4H, 2 × CH_2_), 1.28–1.34 (m, 14H, 7 × CH_2_), 1.18 (d, 3H, *J* = 6.2 Hz, H-6′′′), 1.17 (d, 3H, *J* = 6.2 Hz, H-6′′), 1.16 (d, 3H, *J* = 6.2 Hz, H-6′), 0.90 (t, 3H, *J* = 7.1 Hz, CH_3_), 0.89 (t, 3H, *J* = 7.0 Hz, CH_3_); ^13^C-NMR (CDCl_3_): *δ* 172.9, 171.6, 170.5 (two), 170.3, 170.2, 99.2, 99.0, 97.2, 75.5, 74.5, 74.3, 72.7, 72.2, 72.1, 71.8, 71.7, 68.2, 68.1, 67.4, 66.7, 66.5, 34.0, 31.8, 31.3, 29.3 (two), 29.2, 26.1, 24.5, 22.6, 22.3, 21.1, 21.0, 20.9, 20.8, 17.4, 17.2, 17.1, 14.1, 13.9; HRESIMS calcd for C_42_H_68_O_19_Na [M + Na]^+^ 899.4252; found, 899.4266.

#### 3.2.10. O-Oct-1-yl-α-L-rhamnopyranosyl-(1→3)-α-L-rhamnopyranosyl-(1→3)-α-L-rhamnopyranoside (**10**)

To a stirred solution of **9** (420 mg, 0.51 mmol) in anhydrous methanol (10 mL) sodium methoxide was added (54 mg, 1.0 mmol). The reaction mixture was stirred at at 50 °C for 24 h, after which the reaction mixture was neutralized with Dowex 50 × 8 (H^+^) resin until pH 7, filtered and concentrated in vacuo to furnish a crude product, that was purified via silica gel column chromatography (15:1, trichloromethane—methanol) to yield **10** (0.25 g, 86%); ^1^H-NMR (CD_3_OD): *δ* 5.03 (d, 1H, *J* = 1.6 Hz, H-1′′′), 5.01 (d, 1H, *J* = 1.6 Hz, H-1′′), 4.65 (d, 1H, *J* = 1.7 Hz, H-1′), 4.08 (dd, 1H, *J* = 3.2, 1.8 Hz, H-2′), 3.99 (dd, 1H, *J* = 3.4, 1.7 Hz, H-2′′), 3.88 (dd, 1H, *J* = 3.4, 1.6 Hz, H-2′′′), 3.87 (dd, 1H, *J* = 9.8, 3.3 Hz, H-3′′′), 3.80–3.85 (m, 2H, H-5′′, H-5′′′), 3.79 (dd, 1H, *J* = 9.5, 3.4 Hz, H-3′′), 3.76 (dd, 1H, *J* = 9.6, 3.2 Hz, H-3′), 3.67–3.71 (m, 1H, H-5′), 3.60–3.64 (m, 1H, H-1-1), 3.53 (t, 1H, *J* = 9.5 Hz, H-4′), 3.51 (t, 1H, *J* = 9.6 Hz, H-4′′), 3.41–3.44 (m, 1H, H-1-2), 3.40 (t, 1H, *J* = 9.5 Hz, H-4′′′), 1.58–1.60 (m, 2H, CH_2_), 1.30–1.43 (m, 10H, 5 × CH_2_), 1.28 (d, 3H, *J* = 6.2 Hz, H-6′′′), 1.27 (d, 6H, *J* = 6.2 Hz, H-6′′, H-6′), 0.92 (t, 3H, *J* = 7.1 Hz, CH_3_); ^13^C-NMR (CD_3_OD): *δ* 102.7, 102.5, 100.2, 78.6, 78.0, 72.7, 71.9, 71.8, 70.8, 70.5, 68.9, 68.7 (two), 67.2, 31.6, 29.2, 29.1, 29.0, 26.0, 22.3, 16.6, 13.0; HRMALDIMS calcd for C_26_H_48_O_13_Na 591.2993; found 591.2997.

#### 3.2.11. 1-Octyloxy-2,4-di-*O*-acetyl-3-*O*-(p-methoxybenzyl)-α-l-rhamnopyranosyl-(1→3)-4-*O*-hexanoyl-2-levulinoyl-α-l-rhamnopyranoside (**35**)

To a solution of compound **15** (1.0 g, 2.3 mmol), **34** [20] (1.5 g, 3.2 mmol) and 4 Å molecular sieves in dry CH_2_Cl_2_ (30 mL) *N*-iodosuccinimide (0.90 g, 4.8 mmol) and silver trifluoromethanesulfonate (0.12 g, 0.45 mmol) were added at 0 °C under argon. The reaction mixture was allowed to stir for 2 h under this condition, while warmed to room temperature until TLC indicated that the reaction was complete. Then the residue was diluted with CH_2_Cl_2_ (100 mL), and washed with aqueous Na_2_S_2_O_3_ (50 mL), saturated aqueous NaHCO_3_ (2 × 50 mL), and brine (2 × 50 mL), dried over Na_2_SO_4_, and concentrated in vacuo. The residue was purified by silica gel column chromatography (5:1, petroleum ether–EtOAc) to give a white solid **35** (1.6 g, 92%); ^1^H-NMR (CDCl_3_): *δ* 7.19 (d, 2H, *J* = 8.6 Hz, Ar-H), 6.85 (d, 2H, *J* = 8.6 Hz, Ar-H), 5.11–5.13 (m, 2H, H-2′, H-2′′), 5.05 (t, 1H, *J* = 9.8 Hz, H-4′), 4.96 (t, 1H, *J* = 9.8 Hz, H-4′′), 4.85 (d, 1H, *J* = 1.1 Hz, H-1′′), 4.66 (brs, 1H, H-1′′), 4.53 (d, 1H, *J* = 11.6 Hz, Ar-CH_2_-1), 4.37 (d, 1H, *J* = 11.6 Hz, Ar-CH_2_-2), 4.07 (dd, 1H, *J* = 9.8, 3.2 Hz, H-3′′), 3.79 (s, 3H, OCH_3_), 3.73–3.77 (m, 3H, H-3′, H-5′, H-5′′), 3.61–3.65 (m, 1H, H-1-1), 3.37–3.40 (m, 1H, H-1-2), 2.71 (t, 2H, *J* = 6.3 Hz, COCH_2_), 2.60–2.65 (m, 2H, COCH_2_), 2.36 (t, 2H, *J* = 6.6 Hz, COCH_2_), 2.18, 2.13, 2.02 (each s, each 3H, each CH_3_CO), 1.63–1.66 (m, 2H, CH_2_), 1.54–1.56 (m, 2H, CH_2_), 1.28–1.33 (m, 14H, 7 × CH_2_), 1.19 (d, 3H, *J* = 6.4 Hz, H-6′), 1.14 (d, 3H, *J* = 6.3 Hz, H-6′′), 0.90 (t, 3H, *J* = 7.1 Hz, CH_3_), 0.89 (t, 3H, *J* = 7.2 Hz, CH_3_); ^13^C-NMR (CDCl_3_): *δ* 205.9, 176.5, 173.0, 171.9, 170.2, 159.1, 133.7, 130.1, 129.3 (two), 113.3 (two), 99.8, 97.1, 75.2, 73.8, 72.3, 72.2, 72.0, 71.0, 68.8, 68.1, 67.3, 66.5, 55.2, 37.8, 34.1, 31.8, 31.3, 29.3 (two), 29.2, 28.5, 28.2, 26.1, 24.6, 22.6, 22.3, 20.9, 17.5, 17.3, 14.1, 13.9; HRESIMS calcd for C_43_H_66_O_15_Na [M + Na]^+^ 845.4299; found, 845.4283.

#### 3.2.12. 1-Octyloxy-2,4-di-*O*-acetyl-α-L-rhamnopyranosyl-(1→3)- 4-*O*-hexanoyl-2-levulinoyl-α-l-rhamnopyranoside (**36**)

Reaction of compound **35** (2.0 g, 2.4 mmol), and DDQ (0.72 g, 3.2 mmol) in CH_2_Cl_2_ (60 mL) and H_2_O (4 mL) was essentially as described for **15** yielded **36** (1.6 g, 94%) as a white solid; ^1^H-NMR (CDCl_3_): *δ* 5.12 (dd, 1H, *J* = 3.3, 1.8 Hz, H-2′′), 5.05 (t, 1H, *J* = 9.9 Hz, H-4′), 4.90 (d, 1H, *J* = 1.2 Hz, H-1′′), 4.87 (dd, 1H, *J* = 3.5, 1.6 Hz, H-2′), 4.83 (t, 1H, *J* = 9.8 Hz, H-4′′), 4.66 (d, 1H, *J* = 1.6 Hz, H-1′), 4.07 (dd, 1H, *J* = 10.0, 3.4 Hz, H-3′′), 3.94–3.97 (m, 1H, H-3′), 3.74–3.82 (m, 2H, H-5′, H-5′′), 3.63 (dt, 1H, *J* = 9.5, 2.8 Hz, H-1-1), 3.39 (dt, 1H, *J* = 9.6, 6.6 Hz, H-1-2), 2.70–2.80 (m, 4H, 2 × COCH_2_), 2.46 (dt, 1H, *J* = 16.1, 7.5 Hz, COCH_2_-1), 2.34 (dt, 1H, *J* = 16.0, 7.6 Hz, COCH_2_-2), 2.22, 2.14, 2.13 (each s, each 3H, each CH_3_CO), 1.63–1.66 (m, 2H, CH_2_), 1.54–1.59 (m, 2H, CH_2_), 1.26–1.34 (m, 14H, CH_2_), 1.18 (d, 3H, *J* = 6.4 Hz, H-6′), 1.17 (d, 3H, *J* = 6.3 Hz, H-6′′), 0.90 (t, 3H, *J* = 7.2 Hz, CH_3_), 0.89 (t, 3H, *J* = 7.2 Hz, CH_3_); ^13^C-NMR (CDCl_3_): *δ* 206.1, 173.1, 171.9, 171.5, 170.2, 99.1, 97.1, 74.9, 74.3, 72.9, 72.2, 71.9, 68.1, 67.8, 66.8, 66.6, 37.8, 34.0, 31.8, 31.3, 29.8, 29.3 (two), 29.2, 28.2, 26.1, 24.5, 22.6, 22.3, 21.0, 20.9, 17.5, 17.2, 14.1, 13.9; HRESIMS calcd for C_35_H_58_O_14_Na [M + Na]^+^ 725.3724; found, 725.3730.

#### 3.2.13. 1-Octyloxy-2,4-di-*O*-acetyl-α-l-rhamnopyranosyl-(1→3)-4-O-hexanoyl -α-l-rhamnopyranoside (**11**)

Analogously, **11** was prepared from **36** as described for **2** in 89% yield. ^1^H-NMR (CDCl_3_): *δ* 5.04 (t, 1H, *J* = 9.2 Hz, H-4′), 4.93 (s, 1H, H-1′′), 4.90 (dd, 1H, *J* = 3.2, 1.7 Hz, H-2′′), 4.86 (t, 1H, *J* = 9.7 Hz, H-4′′), 4.75 (s, 1H, H-1′), 4.02 (dd, 1H, *J* = 10.1, 3.1 Hz, H-3′), 3.92–3.97 (m, 3H, H-5′′, H-2′, H-3′′), 3.71–3.79 (m, 1H, H-5′), 3.62–3.67 (m, 1H, H-1-a), 3.37–3.42 (m, 1H, H-1-b), 2.34–2.48 (m, 2H, CH_2_CO), 2.13, 2.12 (each s, each 3H, each CH_3_CO), 1.61–1.66 (m, 2H, CH_2_), 1.53–1.58 (m, 2H, CH_2_), 1.25–1.35 (m, 14H, 7 × CH_2_), 1.20 (d, 3H, *J* = 6.2 Hz, H-6′′), 1.18 (d, 3H, *J* = 6.2 Hz, H-6′), 0.87–0.89 (m, 6H, 2 × CH_3_); ^13^C-NMR (CDCl_3_): *δ* 174.8, 172.8, 171.7, 100.6, 100.4, 79.7, 75.7, 74.2, 73.2, 72.4, 69.5, 69.4, 68.2, 67.6, 35.5, 33.2, 32.7, 30.8, 30.7, 30.6, 27.5, 26.0, 24.1, 23.7, 22.4 (two), 18.8 (two), 15.5, 15.3; HRESIMS calcd for C_30_H_52_O_12_Na [M + Na]^+^ 627.3356; found, 627.3370.

#### 3.2.14. 1-Octyloxy-2,4-di-*O*-acetyl-3-*O*-levulinoyl-α-l-rhamnopyranosyl-(1→3)-2,4-di-*O*-acetyl-α-l-rhamnopyranosyl-(1→3)-2,4-di-*O*-acetyl-α-l-rhamnopyranosyl-(1→3)-4-*O*-hexanoyl-2-*O*-levulinoyl-α-l-rhamnopyranoside (**37**)

Analogously, **37** was prepared from **36**, **24**, and **18** as described for **25** in 80% yield. ^1^H-NMR (CDCl_3_): *δ* 5.14 (dd, 1H, *J* = 10.2, 3.4 Hz, H-3′′′′), 5.08 (dd, 1H, *J* = 3.2, 1.8 Hz, H-2′′′′), 5.04 (t, 2H, *J* = 10.0 Hz, H-4′, H-4′′), 5.03 (t, 2H, *J* = 10.0 Hz, H-4′′′, H-4′′′′), 5.02 (dd, 1H, *J* = 3.2, 2.0 Hz), 4.99 (dd, 1H, *J* = 3.2, 1.8 Hz), 4.94 (d, 1H, *J* = 1.8 Hz), 4.93 (d, 1H, *J* = 3.2, 1.7 Hz), 4.84 (d, 1H, *J* = 1.4 Hz), 4.82 (d, 1H, *J* = 1.1 Hz), 4.66 (d, 1H, *J* = 1.3 Hz), 4.10 (dd, 1H, *J* = 10.0, 3.4 Hz), 4.05 (dd, 1H, *J* = 10.0, 3.3 Hz), 3.93 (dd, 1H, *J* = 10.0, 3.4 Hz), 3.74–3.82 (m, 3H), 3.66–3.70 (m, 1H), 3.61–3.65 (m, 1H, H-1-1), 3.37–3.41 (m, 1H, H-1-2), 2.31–2.79 (m, 10 H, 5 × COCH_2_), 2.22, 2.17, 2.17, 2.16, 2.15, 2.14, 2.12, 2.09 (each s, each 3H, each CH_3_CO), 1.60–1.65 (m, 2H, CH_2_), 1.54–1.58 (m, 2H, CH_2_), 1.26–1.34 (m, 14H, 7 × CH_2_), 1.19 (d, 6H, *J* = 6.2 Hz), 1.16 (d, 3H, *J* = 6.2 Hz), 1.14 (d, 3H, *J* = 6.2 Hz), 0.90 (t, 3H, *J* = 7.2 Hz, CH_3_), 0.89 (t, 3H, *J* = 7.2 Hz, CH_3_); ^13^C-NMR (CDCl_3_): *δ* 206.1, 205.9, 172.9, 171.9, 171.2, 170.4 (two), 170.3, 170.2, 170.1, 99.4, 99.2, 98.8, 75.4, 75.0, 74.8, 72.2, 72.0, 71.8 (two), 71.4, 70.6, 70.0, 68.7, 68.2, 67.3, 67.2, 67.1, 66.5, 37.8, 37.6, 34.0, 31.8, 31.3, 29.7, 29.3 (two), 29.2, 28.2, 27.8, 26.1, 24.5, 22.6, 22.3, 21.0 (two), 20.9 (two), 20.8, 20.7, 17.5, 17.2, 17.1 (two), 14.1, 13.9; HRESIMS calcd for C_60_H_92_O_28_Na [M + Na]^+^ 1283.5673; found, 1283.5690.

#### 3.2.15. 1-Octyloxy-2,4-di-*O*-acetyl-α-l-rhamnopyranosyl-(1→3)-2,4-di-*O*-acetyl-α-l-rhamnopyranosyl-(1→3)-2,4-di-*O*-acetyl-α-l-rhamnopyranosyl-(1→3)-4-*O*-hexanoyl-α-l-rhamnopyranoside (**12**)

Analogously, **12** was prepared from **37** as described for **2** in 85% yield. ^1^H-NMR (CDCl_3_): *δ* 5.06 (t, 2H, *J* = 10.0 Hz, H-4′, H-4′′′′), 5.04 (dd, 1H, *J* = 3.3, 1.2 Hz, H-2′), 5.02 (t, 1H, *J* = 10.0 Hz, H-4′′′), 4.98 (dd, 1H, *J* = 3.3, 1.9 Hz, H-2′′′′), 4.94 (dd, 1H, *J* = 3.3, 1.2 Hz, H-2′′′), 4.89 (d, 1H, *J* = 1.6 Hz, H-1′′′), 4.87 (d, 1H, *J* = 1.2 Hz, H-1′′′′), 4.83 (brs, 1H, H-1′′), 4.81 (t, 1H, *J* = 9.9 Hz, H-4′′′), 4.76 (d, 1H, *J* = 1.1 Hz, H-1′), 4.05 (dd, 1H, *J* = 10.0, 3.3 Hz, H-3′′′′), 3.94 (dd, 1H, *J* = 3.3, 1.2 Hz, H-2′), 3.90–3.93 (m, 3H, H-3′, H-3′′, H-3′′′), 3.85–3.87 (m, 1H, H-5′′), 3.70–3.78 (m, 3H, H-5′, H-5′′′, H-5′′′′), 3.65 (dt, 1H, *J* = 9.5, 2.7 Hz, H-1-1), 3.41 (dt, 1H, *J* = 9.6, 6.5 Hz, H-1-2), 2.31–2.45 (m, 2H, COCH_2_), 2.17, 2.16, 2.15, 2.14, 2.13, 2.12 (each s, each 3H, each CH_3_CO), 1.61–1.65 (m, 2H, CH_2_), 1.55–1.58 (m, 2H, CH_2_), 1.28–1.33 (m, 14H, 7 × CH_2_), 1.21, 1.20, 1.18, 1.17 (each d, each 3H, *J* = 6.4 Hz, H-6′, H-6′′, H-6′′′, H-6′′′′), 0.90 (t, 3H, *J* = 7.2 Hz, CH_3_), 0.89 (t, 3H, *J* = 7.2 Hz, CH_3_); ^13^C-NMR (CDCl_3_): *δ* 173.0, 171.6, 170.4, 170.3 (two), 170.2, 170.1, 99.3 (two), 99.1 (two), 78.8, 75.3, 74.8, 74.3, 72.8, 72.1, 72.0, 71.7 (two), 71.6, 71.0, 68.1, 68.0, 67.5, 67.3, 66.7, 66.2, 34.1, 31.8, 31.3, 29.4, 29.3, 29.2, 26.1, 24.5, 22.6, 22.2, 21.0, 20.9 (three), 20.8, 20.7, 17.4 (two), 17.1, 17.0, 14.0, 13.9; HRESIMS calcd for C_50_H_80_O_24_Na [M + Na]^+^ 1087.4937; found, 1087.4946.

#### 3.2.16. 1-Octyloxy-α-l-rhamnopyranosyl-(1→3)-α-l-rhamnopyranosyl-(1→3)-α-l-rhamnopyranosyl-(1→3)-4-*O*-hexanoyl-α-l-rhamnopyranoside (**13**)

To a stirred solution of **37** (320 mg, 14.9 mmol) in dry CH_3_OH (10 mL) MeONa (65 mg, 1.2 mmol) was added at 0 °C. The reaction mixture was stirred at 0 °C for 2 h, after which the reaction mixture was neutralized with Dowex 50 × 8 (H^+^) resin until pH 7, filtered and concentrated in vacuo to furnish a crude product, which was purified via silica gel column chromatography (8:1, trichloromethane—methanol) to give **13** (185 mg, 82%) as a white solid. ^1^H-NMR (CD_3_OD): *δ* 5.11 (t, 1H, *J* = 10.0 Hz, H-4′), 5.03 (d, 1H, *J* = 1.6 Hz, H-1′′′′), 5.00 (d, 1H, *J* = 1.6 Hz, H-1′′′), 4.78 (d, 1H, *J* = 1.3 Hz, H-1′′), 4.69 (d, 1H, *J* = 1.6 Hz, H-1′), 4.08 (dd, 1H, *J* = 3.2, 1.8 Hz, H-2′′′), 3.99 (dd, 1H, *J* = 3.4, 1.7 Hz, H-2′′), 3.93 (dd, 1H, *J* = 3.2, 1.7 Hz, H-2′), 3.89 (dd, 1H, *J* = 9.8, 3.2 Hz, H-3′), 3.87 (dd, 1H, *J* = 9.6, 3.3 Hz, H-3′′′′), 3.76–3.85 (m, 7H, H-4′′′, H-4′′, H-2′′′′, H-3′′′, H-3′′, H-5′, H-5′′′′), 3.68–3.72 (m, 1H, H-1-1), 3.47–3.54 (m, 2H, H-5′′′, H-5′′), 3.43–3.47 (m, 1H, H-1-2), 3.40 (t, 1H, *J* = 9.5 Hz, H-4′′′′), 2.37 (td, 2H, *J* = 7.5, 1.7 Hz, COCH_2_), 1.59–1.67 (m, 4H, 2 × CH_2_), 1.32–1.44 (m, 14H, 7 × CH_2_), 1.28 (d, 6H, *J* = 6.2 Hz, H-6′, H-6′′), 1.27 (d, 3H, *J* = 6.2 Hz, H-6′′′), 1.15 (d, 3H, *J* = 6.2 Hz, H-6′′′′), 0.93 (t, 3H, *J* = 6.9 Hz, CH_3_), 0.92 (t, 3H, *J* = 7.0 Hz, CH_3_); ^13^C-NMR (CD_3_OD): *δ* 172.9, 102.7, 102.6, 102.5, 100.1, 78.6, 77.8, 77.2, 72.7, 72.5, 71.8, 71.7, 70.8 (three), 70.7, 70.5, 69.2, 68.9, 68.7, 67.4, 66.5. 33.8, 31.6, 31.1, 29.1, 29.0 (two), 25.9, 24.3, 22.3, 21.9, 16.5 (three), 16.4, 13.0, 12.9; HRESIMS calcd for C_38_H_68_O_18_Na [M + Na]^+^ 835.4303; found, 835.4306.

#### 3.2.17. 1-Octyloxy-α-L-rhamnopyranosyl-(1→3)-α-L-rhamnopyranosyl-(1→3)-α-L-rhamnopyranosyl-(1→3)-α-L-rhamnopyranoside (**14**)

To a stirred solution of **37** (320 mg, 14.9 mmol) in dry CH_3_OH (10 mL) MeONa (81 mg, 1.5 mmol) was added at 50 °C. The reaction mixture was stirred at 50 °C for 24 h, after which the reaction mixture was neutralized with Dowex 50 × 8 (H^+^) resin until pH 7, filtered and concentrated in vacuo to furnish a crude product, which was purified via silica gel column chromatography (4:1, trichloromethane—methanol) to afford **14** (180 mg, 91%) as a white solid. ^1^H-NMR (CD_3_OD): *δ* 5.03 (d, 1H, *J* = 1.7 Hz, H-1′′′′), 5.02 (d, 1H, *J* = 1.6 Hz, H-1′′′), 5.01 (d, 1H, *J* = 1.3 Hz, H-1′′), 4.65 (d, 1H, *J* = 1.7 Hz, H-1′), 4.07–4.09 (m, 2H), 3.99 (dd, 1H, *J* = 3.4, 1.7 Hz, H-2′′), 3.86–3.90 (m, 4H), 3.80–3.85 (m, 2H), 3.79 (dd, 1H, *J* = 9.5, 3.4 Hz), 3.76 (dd, 1H, *J* = 9.6, 3.3 Hz), 3.67–3.71 (m, 1H, H-1-1), 3.60–3.65 (m, 1H), 3.50–3.56 (m, 3H), 3.39–3.44 (m, 2H), 1.58–1.63 (m, 2H, H-2), 1.35–1.42 (m, 2H, H-3), 1.32–1.35 (m, 8H, 4 × CH_2_), 1.28 (d, 6H, *J* = 6.2 Hz, H-6′, H-6′′), 1.27 (d, 6H, *J* = 6.2 Hz, H-6′′′, 6′′′′), 0.92 (t, 3H, *J* = 7.2 Hz, CH_3_); ^13^C-NMR (CD_3_OD): *δ* 106.6, 106.5, 106.4, 104.1, 82.5, 82.4, 82.0, 76.7, 75.8 (three), 74.8 (two), 74.7, 74.5 (two), 72.9 (two), 72.7 (two), 71.2, 35.5, 33.1, 33.0, 32.9, 29.9, 26.2, 20.5, 16.9; HRMALDIMS calcd for C_32_H_58_O_17_Na 737.3572; found 737.3576.

### 3.3. Aphicidal Bioassay

The aphicidal activity of the mezzettiasides **2**–**14** against *Aphis glycines* was determined using the reported procedure [24,25]. Compounds were dissolved to a concentration of 2000 mmol/L and then diluted to lower concentrations with water containing 0.05% Triton X-100. Soybean leaf discs of about 3 cm diameter were dipped into the test solution for 15 s. The discs dipped into 0.05% Triton X-100 were set as the negative control. After air-drying, the treated leaf discs were placed individually into bioassay plates with 1% agar to keep moist. The discs were infested with 20 ± 3 three-day old aphids and kept in an incubator with constant temperature (25 ± 1 °C) for 48 h. The number of dead aphids was then counted, and the mortality rates were corrected by use of Abbott’s formula [26]. Each experiment was performed three times. The standard deviations of the tested aphicidal values were ≤10%. The LC_50_ values were calculated using the EXCEL.

## 4. Conclusions

In summary, a series of novel oligo-rhamnoside compounds with biosurfactant characteristics were designed and synthesized. The bioassay results indicated that most of the title compounds exhibited the considerable aphicidal activity against *A. glycines* in the laboratory. In particular, oligo-rhamnoside **9** showed higher aphicidal activity than pymetrozine, with an LC_50_ of 0.019 mmol/L, suggesting that it may be used as a potential lead compound for the discovery of potential eco-friendly insecticides. The preliminary SARs of the oligo-rhamnoside analogues indicated that the degree and location of acylation on the different alcoholic hydroxyl groups had important influence on the aphicidal activity and the disaccharide residue 1-*O*-n-Octyl- -*α*-l-rhamnopyranosyl-(1→3)-*α*-l-rhamnopyranoside played a key role in bioactivity. However, further research on acute toxicity, field residues, and their insecticidal mode of action is ongoing and will be reported in the future. Since no data were obtained in this study on selectivity against Aphids, it will be of high importance to determine the toxicity of the compounds under study against beneficial insects.

## Figures and Tables

**Table 1:** Aphicidal activity against *Aphis glycines* of target compounds **2**–**14.**

Compound	Mortality (%) (100 mmol·L^−1^, 48 h)	^a^ y = a + bx	^b^ LC_50_ mmol·L^−1^	R	^c^ 95% FL
**2**	96.82 ± 1.12	y = 8.38 + 2.03x	0.022	0.99	0.012–0.039
**3**	82.45 ± 1.72	y = 7.89 + 2.21x	0.049	0.99	0.031–0.077
**4**	79.67 ± 2.35	y = 7.59 + 2.10x	0.058	0.97	0.036–0.095
**5**	80.32 ± 2.62	y = 7.56 + 1.77x	0.036	0.98	0.020–0.062
**6**	47.88 ± 1.82	-	^d^ NT	-	-
**7**	95.62 ± 2.64	y = 8.71 + 2.31x	0.025	0.99	0.015–0.041
**8**	83.58 ± 1.63	y = 7.59 + 1.98x	0.049	0.97	0.030–0.081
**9**	98.95 ± 1.38	y = 8.59 + 2.09x	0.019	0.98	0.010–0.035
**10**	25.12 ± 1.52	-	NT	-	-
**11**	92.82 ± 2.26	y = 8.17 + 2.16x	0.034	0.99	0.020–0.057
**12**	78.68 ± 2.45	y = 7.85 + 2.11x	0.045	0.97	0.027–0.073
**13**	28.12 ± 1.43	-	NT	-	-
**14**	20.56 ± 1.15	-	NT	-	-
pymetrozine	99.25 ± 2.52	y = 7.25 + 1.68x	0.032	0.98	0.012–0.080

^a^ This equation represent the toxicological regression line, and it was arrived in Excel based on the principle of probit analysis according to Liu’s work [23]. Where “y” and “x” are mean values, and “a” and “b” means “intercept” and “slope”, respectively. The process of details calculation is listed in the following formula: P (Corrected mortality) = (Treatment Mortality − Control Mortality)/(1 − Control Mortality) × 100%; Z (Weight coefficient parameter) = (2π^−1/2^) × e^−(Y − 5)/2^. (“Y” is the mortality probability value); W (Weight coefficient) = Z^2^/P (1 − P); y = Σ*_nwy_*/Σ*_nw_*; x = Σ*_nwx_*/Σ*_nw_*; Slope b = (Σ*_nw_* × Σ*_nwxy_* − Σ*_nwx_* × Σ*_nwy_*)/[Σ*_nw_* × Σ*_nwx_*^2^ − (Σ*_nwx_*) ^2^]; Intercept a = y − bx. ^b^ LC_50_ value was not tested when screening mortality was lower than 50% at the concentration of 100 mmol·L^−1^, 48 h. ^c^ 95% FL: 95% fiducial interval; ^d^ NT: not test.
